# Tracing the cellular basis of islet specification in mouse pancreas

**DOI:** 10.1038/s41467-020-18837-3

**Published:** 2020-10-07

**Authors:** Magdalena K. Sznurkowska, Edouard Hannezo, Roberta Azzarelli, Lemonia Chatzeli, Tatsuro Ikeda, Shosei Yoshida, Anna Philpott, Benjamin D. Simons

**Affiliations:** 1 Department of Oncology, University of Cambridge, Hutchison/MRC Research Centre, Cambridge Biomedical Campus, Cambridge, CB2 0XZ UK; 2 Wellcome Trust/Medical Research Council Cambridge Stem Cell Institute, University of Cambridge, Tennis Court Road, Cambridge, CB2 1QR UK; 3 IST Austria, Am Campus 1, 3400 Klosterneuburg, Austria; 4 The Wellcome Trust/Cancer Research UK Gurdon Institute, University of Cambridge, Tennis Court Road, Cambridge, CB2 1QN UK; 5 Division of Germ Cell Biology, National Institute for Basic Biology, National Institutes of Natural Sciences, 5-1 Higashiyama, Myodaiji, Okazaki, 444-8787 Japan; 6 Department of Basic Biology, School of Life Science, Graduate University for Advanced Studies (Sokendai), 5-1 Higashiyama, Myodaiji, Okazaki, 444-8787 Japan; 7 AMED-CREST, Japan Agency for Medical Research and Development, Tokyo, 100-0004 Japan; 8 Department of Applied Mathematics and Theoretical Physics, Centre for Mathematical Sciences, University of Cambridge, Wilberforce Road, Cambridge, CB3 0WA UK

**Keywords:** Computational models, Differentiation, Organogenesis

## Abstract

Pancreatic islets play an essential role in regulating blood glucose level. Although the molecular pathways underlying islet cell differentiation are beginning to be resolved, the cellular basis of islet morphogenesis and fate allocation remain unclear. By combining unbiased and targeted lineage tracing, we address the events leading to islet formation in the mouse. From the statistical analysis of clones induced at multiple embryonic timepoints, here we show that, during the secondary transition, islet formation involves the aggregation of multiple equipotent endocrine progenitors that transition from a phase of stochastic amplification by cell division into a phase of sublineage restriction and limited islet fission. Together, these results explain quantitatively the heterogeneous size distribution and degree of polyclonality of maturing islets, as well as dispersion of progenitors within and between islets. Further, our results show that, during the secondary transition, α- and β-cells are generated in a contemporary manner. Together, these findings provide insight into the cellular basis of islet development.

## Abstract (web summary)

The cellular basis of islet morphogenesis and fate allocation remain unclear. Here, the authors use a R26-CreER-R26R-Confetti mouse line to follow quantitatively the clonal dynamics of islet formation showing how, during the secondary transition, islet progenitors amplify through rounds of stochastic cell division before becoming restricted to α and β cell sublineages.

## Introduction

**Fig. 1:**
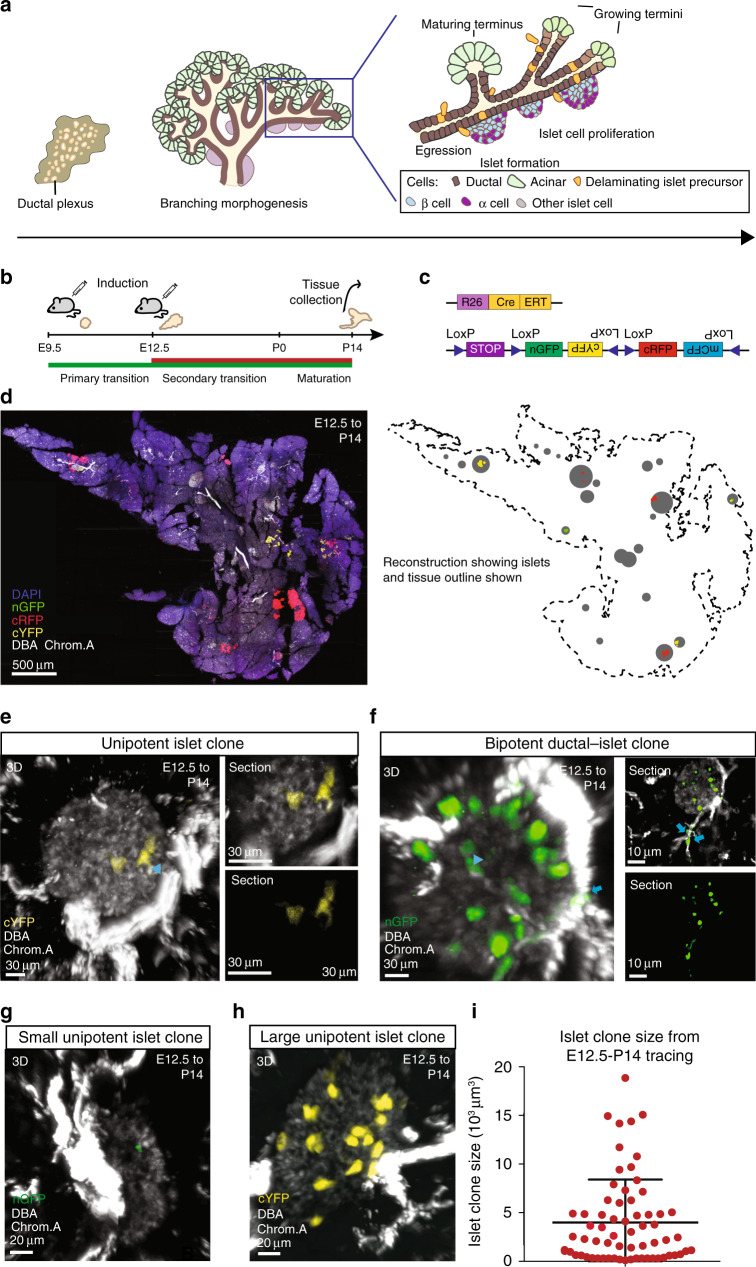
Lineage tracing reveals broad distribution of islet clone sizes. **a** Schematic representing pancreas development, from plexus formation, through a phase of branching morphogenesis and islet formation. Islets are formed by the egression of endocrine progenitors from the ductal epithelium. They aggregate together to form a mantle–core structure, composed of insulin+ β-cells surrounded by glucagon+ α-cells, as well as cells from other rarer endocrine lineages. **b** Schematic representing the experimental schedule, with a single injection of TAM at either E9.5 or E12.5 and tissue collection at P14. **c** *R26-CreERT2/R26R-Confetti* labelling construct. **d** A 100 μm pancreatic section from *R26-CreERT2/R26R-Confetti* mice induced at E12.5 and fixed at P14 with islets immunostained by Chromogranin A (grey) and ducts stained by DBA (white) (left panel) showing a low fraction of labelled islets. The reconstruction (right panel) depicts the corresponding tissue outline, as well as the position of labelled and non-labelled islets. **e**, **f** Examples of unipotent (**e**) and bipotent (**f**) clones. For the relative abundances of different clone potencies, see ref. ^6^. The size of the islet compartment of all the traced clones were characterised by a wide distribution from **g** small clones of 1–3 cells to **h** large clones (≥15 cells). Chromogranin A is shown in grey and DBA in white. **d**–**h** are respectively representative of >15,>20, >20, >20 and 5 recorded images from 3 experiments each. **i** Sizes of individual islet clones from the E12.5 to P14 tracings, defined as the total volume of labelled islet cells within individual tri-, bi- and unipotent clones (*n* = 55 clones from *N* = 3 mice), showing a broad distribution. Error bars show average ± SD. Source data provided as Source Data file.

Hormone secretion by the islets of Langerhans in the pancreas regulates blood glucose level, while abnormal islet function leads to diabetes mellitus^1^. Mature islets in mice are comprised predominantly of an external mantle of glucagon producing α-cells and an internal core of insulin producing β-cells. They also contain minority populations of cells secreting other pancreatic hormones such as somatostatin, ghrelin and Polypeptide P (Fig. 1a). Based on their central role in regulating glucose homoeostasis, much emphasis in pancreas biology has been placed on resolving the signalling pathways involved in the generation of β-cells during development^2,3^, and their maintenance in adulthood^4,5^.

Previously, we and others have shown that, following an early phase of plexus remodelling, the large-scale organisation of mouse pancreas involves an extended phase of ductal branching morphogenesis, driven by minority subpopulations of sublineage-restricted, self-renewing progenitors that localise at the termini of ducts^6–8^. One population drives rounds of ductal bifurcation, giving rise to unipotent (ductal) and bipotent (ductal–islet) progenitors, while a second population of acinar-committed progenitors travels with cells at the ductal termini, replicating during tip bifurcation and finally differentiating into acinar cells^6^. Following commitment to the islet lineage, progenitors egress from the ductal epithelium^9^. It has been proposed that the central plexus, which remodels during embryonic development, may provide a niche for endocrine cell differentiation, with robust, non-autonomous and long-lived production of islet progenitors occurring until late embryogenesis^7^. A lineage tracing study has shown that progenitors with an active promoter of *Neurogenin3* (*Ngn3*), the basic helix–loop–helix (bHLH) transcription factor and master regulator of endocrine differentiation, are restricted to islet sublineages, and undergo few rounds of division between birth and adulthood^10^, with further compensatory replication and maturation acquired after weaning^11^. However, the timing, potency and growth potential of individual islet progenitors during normal embryonic development remains poorly defined.

Based on lineage tracing and gene knock out studies, it is known that islets arise from cells in maturing embryonic pancreatic ducts that express *Ngn3*^12–15^. Individual islets in the adult pancreas are also known to be polyclonal in origin^16^. Recently, transcriptomic analyses and 3D imaging of the embryonic day (E)13.5–E14.5 pancreas suggested that a temporally graded differentiation of *Ngn3*+ progenitors, occurring first into the α and then the β-cell sublineage, could explain the mantle–core structure of mature islets^17^. However, whether this temporal gradient in α- and β-cell production persists during later stages of development remains unclear. It is also unclear whether fate allocation to the endocrine lineage and its sublineages occur stochastically and where, spatially, commitment occurs. Finally, in adult, both mouse and human islets are found to be highly heterogeneous in size, with variations of up to a thousandfold in volume^18,19^. While islet fusion and fission processes have been suggested to underlie this heterogeneity^18,19^, their cellular and mechanistic basis remain unknown.

Here, we combine unbiased and targeted genetic lineage tracing strategies using mouse models based on a *Rosa26 (R26)* and *Ngn3* promoter, respectively, with three-dimensional confocal imaging and mathematical modelling to address cell fate behaviour, sublineage restriction and spatial patterning during islet morphogenesis in the mouse pancreas. In particular, we show that, during the secondary transition, islet formation involves the aggregation of multiple equipotent endocrine progenitors that expand by stochastic proliferation after which they enter a phase of sublineage restriction and limited islet fission. Together, these findings provide a quantitative explanation for the heterogeneous size distribution and degree of polyclonality of maturing islets, as well as dispersion of clones within and between islets.

## Results

### Unbiased lineage tracing of islet progenitors

To address the dynamics of islet development, we used the *R26-CreERT2/R26R-Confetti* mouse model to trace the fate of progenitors in the embryonic pancreas. Using the *R26R-Confetti* mouse line, four fluorescent reporter genes (GFP, YFP, RFP and CFP) can be expressed at random after Cre‐mediated recombination, providing a hereditary mark that records the fate of induced cells and their progenies. By linking Cre expression to the ubiquitous *R26* promoter, the labelling strategy is able to activate a fluorescent reporter in any cell type in an unbiased manner. Recently, we have used this model to investigate the cellular dynamics underlying the large-scale spatio-temporal patterning of the mouse pancreas, with a focus on the specification of the ductal and acinar compartments^6^.

To achieve clonal induction, a low dose of Tamoxifen (TAM) was administered to mice resulting in sparse labelling of tissue (<3% by volume) at the start of the two key stages of pancreatic development corresponding to the onset of the primary and secondary transition^20,21^; E9.5 and E12.5 (Fig. 1b–d). Based on the reported time-delay between TAM administration and induction for Cre-ERT2^22^, cells may be marked up to 24 h post injection. To target islet development, we quantified the islet cell content of individual clones at postnatal day (P)14, when commitment of cells to the pancreatic sublineages is thought to be complete^20^, using 3D tissue reconstructions derived from thick serial sections stained for the islet marker Chromogranin A (with 48 clones reconstructed from *n* = 3 mice for E9.5 induction and 55 clones from *n* = 3 mice for E12.5; “Methods”). Notably, we found that, where clones comprised both islet and ductal cells, the corresponding islet and duct were in close proximity, consistent with reports of local egression of endocrine cells^16,17^.

At the given level of induction, most P14 islets (92% from E9.5 and 82% from E12.5) were comprised exclusively of unlabelled cells, while the majority of labelled islets contained cells sharing the same confetti colour (82% and 72% of labelled islets from E9.5 and E12.5 tracings, respectively) (Fig. 1d and Supplementary Fig. 1). Such labelling events could arise through the chance induction of different progenitors that give rise to progenies that occupy the same islet. Fortunately, by using a confetti labelling system, we could use measurements of the colour mosaicism of individual islets to estimate the frequency at which independent clones of the same colour could arise by chance in the same islet (for details, see “Methods” Statistical inference of clonality). Accordingly, we found that, overall, <10% of cell clusters labelled by a common colour in a given islet were likely to be the result of chance clone merger.

Similarly, we assessed whether an individual labelled progenitor could give rise to progenies that spanned multiple neighbouring islets at P14. Once again, quantitative analysis of neighbouring islets bearing cells of a common colour suggested that the frequency of such events was low, with only 15% of clones from the E12.5 induction extending across multiple neighbouring islets compared to 37% from E9.5 (“Methods” Statistical inference of clonality). It followed that the majority of labelled cell clusters within individual islets were of clonal origin, and represent the full islet cell output of an individual tri-, bi- or unipotent progenitor labelled at E9.5 or E12.5 (Fig. 1e, f and Supplementary Fig. 1a). (Note that, here, we define a tripotent islet clone as the islet cell contribution of a clone containing ductal, acinar and islet cells; a bipotent islet clone as the islet cell contribution of a clone containing ductal and islet cells, or—although much rarer—acinar and islet cells; and a unipotent islet clone as one containing only islet cells.) Based on this assignment, we then quantified the islet cell number of individual islet clones (spanning either a single islet or a few neighbouring islets) from the two induction times.

### Islet progenitors aggregate and proliferate stochastically

**Fig. 2:**
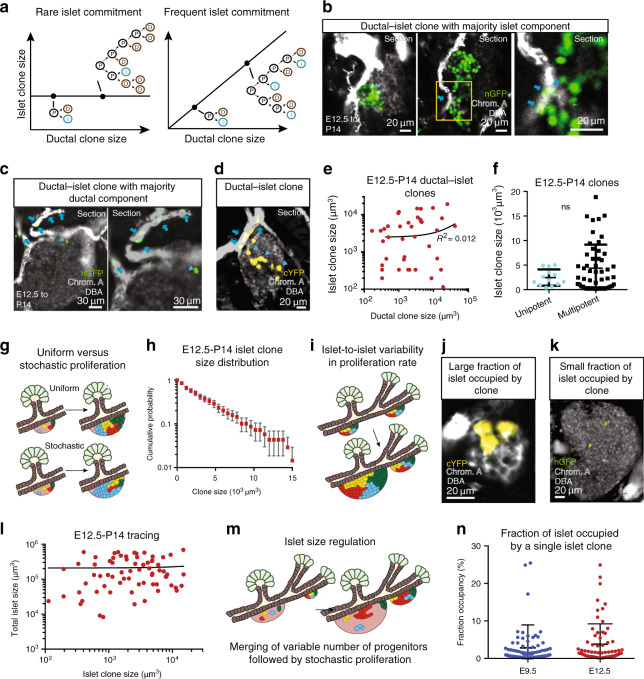
Coalescence and stochastic proliferation of islet progenitors underlies islet size heterogeneity. **a** (Left) Schematic depicting specification of islet progenitors from bipotent ductal–islet progenitors: If islet progenitor production were a rare event, islet clone size would be independent of ductal clone size. If the rate were high, islet clone size would scale in approximate proportion to ductal clone size. Representative images from 3 experiments based on >15 recorded images each showing examples of ductal–islet clones with islet representation being **b** majority, **c** minority and **d** approximately equal. **b**, **c** show snapshots from different Z-sections. Arrows indicate ductal cells, arrowheads indicate islet cells. DBA shown white, Chromogranin A in grey. **e** Dot plot of ductal to islet size within individual clones showing weak correlation (*R*^2^ = 0.012). **f** Plot of islet size of unipotent (islet-only) and multipotent clones showing no statistically significant difference in average size (*n* = 13 unipotent and *n* = 53 multipotent clones from *N* = 3 mice, *P* = 0.76, two-tailed Mann–Whitney test). Error bars show average ± SD. **g** Schematic depicting possible modes of islet expansion: (top) endocrine progenitors expand uniformly within islets; (bottom) progenitors have variable proliferative capacity. **h** Cumulative distribution of islet clone sizes shows exponential-like dependence (“Methods”, *n* = 69 clones from *N* = 3 mice). Error bars show average ± SD. **i** Schematic depicting hypothesis that islet size heterogeneity is associated with local islet-to-islet variability in proliferative activity of constituent progenitors. Representative images (based on >10 recorded images each) from 3 experiments showing variable labelling fraction within islets with **j** large clone fraction and **k** small clone fraction. DBA shown white, Chromogranin A in grey. **l** Dot plot showing that there is no correlation between islet clone size and total size of host islet (*R*^2^ = 0.001). **m** Summary schematic of islet growth whereby the final size of islets is dictated by degree of polyclonality arising from coalescence of islet progenitors combined with stochastic proliferation. Smaller islets are, on average, composed of a smaller number of founding progenitors that merge. **n** Fraction of islets occupied by individual islet clones traced from E9.5 to P14 and E12.5 to P14 (*n* = 93 clones from *N* = 5 mice at E9.5 and *n* = 69 clones from *N* = 3 mice at E12.5). Error bars show average ± SD. Source data provided as Source Data file.

When quantified, we found a large degree of islet clone size variability (Fig. 1g–i and Supplementary Fig. 1a–j), with some clones containing tens of islet cells while others contained as few as one. We first considered whether the origin of size variability could derive from differences in the rate at which progenitors become specified towards the endocrine lineage. This would be consistent with reports that, during development, the allocation of islet fate occurs in a highly asynchronous manner^17^, so that the net islet cell output of individual progenitors may depend on their prior amplification. We reasoned that, if the source of islet clone size heterogeneity was associated with late specification towards the islet lineage, leading to the frequent production of multiple endocrine progenitors then, for clones containing both ductal and islet components, the average islet clone size should scale roughly in proportion to the size of the ductal compartment. If, on the other hand, separation of the ductal/endocrine lineages occur early, then islet specification would be a rare event, and the size of the two compartments in multipotent clones should be statistically uncorrelated (Fig. 2a).

We therefore examined the correlation between the ductal and islet compartments within the same clone, combining both bipotent (ductal–islet) clones and tripotent clones. The comparison failed to reveal significant correlation from either the E12.5 (*R*^2^ = 0.012, Fig. 2b–e) or E9.5 tracings (*R*^2^ = 0.06, Supplementary Fig. 1i). Indeed, further comparison of islet clone size between unipotent and multipotent clones indicated that the potential size advantage of the latter was small, and not statistically significant (*P* = 0.76 for E12.5 clones, Fig. 2f, Mann–Whitney test). Together, these results suggest that, for the majority of islet clones, their size represents the total output of individual progenitors following commitment to the endocrine lineage. (Note that, here, we use “commitment” to indicate the point at which a progenitor becomes fated towards the endocrine lineage under unperturbed conditions. We cannot rule out the possibility that, if environmental conditions were no longer permissive, progenitors could acquire a different fate.)

We then questioned whether the predominant source of size variability could originate from heterogeneity in the proliferative activity of progenitors following commitment to the islet lineage (Fig. 2g). Previous studies have shown that insight into the potency and fate behaviour of progenitors can be gained from the distribution of clone sizes^23^. Strikingly, statistical analysis showed that the distribution of islet clone size at both induction times was consistent with an exponential size dependence (Fig. 2h and Supplementary Fig. 1j, *P* > 0.2 from a Kolmogorov–Smirnoff test; “Methods” Theoretical basis for interpreting lineage tracing datasets). Such behaviour is a hallmark of a single equipotent population of progenitors undergoing serial rounds of stochastic cell division^24^. Here, by stochastic, we mean probabilistic, with the timings between consecutive cell divisions statistically uncorrelated, and drawn at random from an exponential distribution with a given average cell cycle time. Alternative cellular hierarchies involving, for example, two or more endocrine progenitor populations would be manifest in the emergence of more complex, multimodal distributions (see “Methods” Alternative models and parameter-fitting). Such behaviour echoes the results of clonal fate studies of the adult mouse pancreas, which suggest that islet cells are renewed through stochastic self-duplication rather than the activity of a minority stem cell-like pool (Fig. 2g, Supplementary Fig. 1n)^25^.

We then investigated whether the size of clonally labelled cell clusters within individual islets were correlated with the total size of the host islet. We reasoned that, on commitment to the endocrine lineage, progenitors might harbour the same proliferative potential, but that local factors could cause some progenitors to proliferate more than others so that large islets host the largest clonal clusters (Fig. 2i). Yet, despite large variations in islet size (>30-fold for the ensemble of islets containing labelled cells), there was little correlation between islet and clone size, either from E9.5 or E12.5 tracings (*R*^2^ = 0.001 and *R*^2^ = 0.001, respectively; Fig. 2j–l and Supplementary Fig. 1k; “Methods”). This suggested that the proliferative behaviour of islet progenitors is not variable between islets, ruling out, for example, the possibility that large islets arise through locally enhanced proliferation.

### Progressive transition from fusion to fission

We then questioned the origin of the size heterogeneity of islets themselves. From the findings above, it follows that variability in islet size is not derived from coordinated proliferative activity of islet cells. Instead, it must originate from variations in the net number of progenitors that associate to form individual islets (Fig. 2m) (“Methods” Number of progenitors per islet). Quantitatively, we found that the average fraction *f* of clonally labelled cells in a given islet is very small at P14, both from E9.5 (*f* = 2.7 ± 0.7%, mean ± SEM) and E12.5 (*f* = 3.5 ± 0.8%, mean ± SEM) tracings (Fig. 2n). It followed that the effective number *N* = 1/*f* of progenitors that found an islet is around *N* = 28 ± 6 (mean ± SEM) on average from the E12.5 tracing and *N* = 37 ± 10 from E9.5. This argues that islets develop as highly polyclonal compound structures (Fig. 2m).

**Fig. 3:**
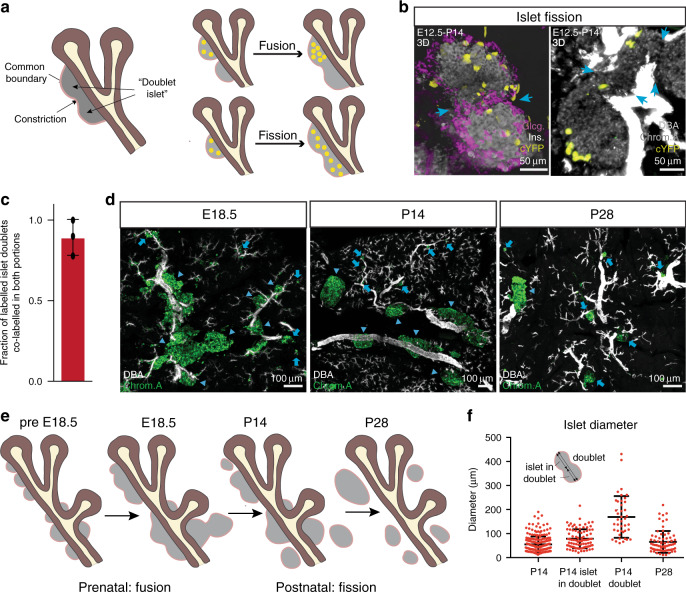
Fission predominates at post-natal stages. **a** (Left) Schematic of “islet doublets”, defined as nascent islets sharing a common boundary separated by a narrowing or isthmus. If islet doublets form through islet fusion, islet clones (yellow) are expected to reside in only one portion of the doublet (top right). If doublets form through fission, islet clones are expected to span both portions (bottom right). **b** Example images from 3 experiments (based on >40 recorded images) of putative islet fission events showing the spreading of the clone into both portions of a doublet. Left panel shows staining for insulin (grey) and glucagon (pink), and right panel shows staining for Chromogranin A (grey) and DBA (white). Arrows indicate constriction between two doublet islets. **c** Fraction of all doublets co-labelled by the same confetti colour in each portion (*n* = 33 islets from *N* = 3 mice). Error bars show average ± SD. **d** 3D projections of Chromogranin A (grey) and DBA (white) stained tissue reveal the evolution of islet structure and location. Small and large islets indicated with arrows and arrowheads, respectively. Representative of 3 experiments (based on >10 images). **e** Schematic summarising the key events during islet formation (cf. panel **d**). **f** Quantification of diameters of single and doublet islets as follows: P14—diameter of single islets at P14; P14 islet in doublet—diameter of constituent islets in a doublet at P14; P14 doublet—diameter of islet doublet; P28—diameter of single islet at P28. For single islets, the diameter along the longest axis was recorded. For islet doublets, diameters were recorded along the longest axis of the doublet, as indicated in the inset of the panel (*n* = 270 islets including 40 doublets from *N* = 3 mice for P14; *n* = 71 islets from *N* = 3 mice for P28). Error bars show average ± SD. Source data provided as Source Data file.

We then questioned whether the spatial organisation of clones could provide insight into the dynamics of islet formation. Recently, it has been shown that, following egression, islet progenitors initially stay associated with ducts^17^ (Fig. 1a). During their subsequent expansion, it has been proposed that newly formed, or nascent, islets may continue to fuse or undergo fission, influencing the final islet size. Therefore, to probe the dynamics of islet formation, we focused on the ensemble of “islet doublets” at P14, where two islets are joined by a narrowing or isthmus (Fig. 3a, left)^26,27^. We reasoned that the distribution of clonal “footprints” on such doublets could discriminate between putative fission or fusion-type processes^27,28^, as illustrated by an analogous study of intestinal crypt fusion/fission^29^. Specifically, at clonal density, we reasoned that islet fusion should lead to events in which, typically, only one portion of a doublet contains labelled cells; by contrast, following cell dispersion during islet expansion (Fig. 1e–h), fission should result in the co-labelling of both portions of a doublet (Fig. 3a, b).

Strikingly, from the analysis of 33 doublets containing labelled cells from the E12.5–P14 tracings (*n* = 4 mice), 84% contained the same confetti colour in both portions, suggesting that islet fission predominates over fusion (Fig. 3c). However, as few clones spanned neighbouring non-doublet islets (15% of E12.5–P14 islet clones), we deduced that such fission events must be relatively rare, at least before P14.

To assess the timing of fission events, we examined *R26-CreERT2*/*R26R-Confetti* tracings from E12.5 to E18.5. At E18.5, islets were arranged in the manner of “beads on a string” in which nascent islets were associated closely, becoming resolved into more separated structures only later in development (Fig. 3d, e and Supplementary Fig. 1m). At this timepoint, we found a lower percentage of islet doublets with co-labelled portions (50%), suggesting that fusion is more prominent at earlier stages (Supplementary Fig. 1l). This result was consistent with the high degree of islet polyclonality^16^, and suggested that islet formation involves a condensation process in which local egression and subsequent proliferation of islet progenitors is accompanied by the fusion of nascent islets^17^, followed by a low rate of fission during neonatal growth (Fig. 3d, e).

To understand whether there might be an intrinsic size-dependent mechanism driving islet fission, we investigated the total size of islet doublets and the size of constituent islets, as well as single (isolated) islets. While the sizes of single islets vary, the average size of an islet at P14 and P28 showed no statistically significant difference (*P* > 0.1, Mann–Whitney). However, strikingly, the average size of islet doublets was almost double the average size of single islets, suggesting that fission events are correlated with islet size. Indeed, if fission occurred randomly in islets of any size, the overall average size of a doublet undergoing fission would be the same as the average size of single islets (Fig. 3f).

### Stochastic fate allocation of α- and β-cell restriction

So far, we have focussed on lineage segregation between the ductal and islet compartments. However, islet cells become further segregated into sub-compartments: In adult, insulin-producing β-cells comprise the majority of islet cells in mouse (65–90%), while glucagon-producing α-cells, which localise on the islet periphery, make up the other significant compartment (5–20%)^30,31^. Notably, this organisation contrasts with human, where islet composition is more variable with β-cells (30–80%) and α-cells (10–65%) intermingling in a more “salt-and-pepper”-like arrangement^30,31^. Lineage tracing studies using a mosaic analysis with double-markers (MADM) mouse reporter system based on constitutive Cre driven from the *cis*-regulatory regions in a *Ngn3-Cre* transgene have argued in favour of early (embryonic) sublineage restriction of islet progenitors into α- and β-cell compartments^10^. However, by the nature of the assay, where the timing of induction is uncontrolled, the timing of sublineage restriction is impossible to infer. Further, a study based on the activation of *Ngn3* expression, introduced exogenously from a transgene construct in *Ngn3* knock out mice, has examined the competency of endocrine cells to form different hormone-producing cells at different developmental stages^32^. Here, it was argued that the competence of pancreatic progenitors to produce non-glucagon+ cell types is acquired during mid-gestation/later periods in organogenesis. However, a detailed map of the dynamics of fate allocation in wild-type animals is still lacking. In particular, the timing and pattern of sublineage restriction of islet progenitors remain unclear, as does the relative growth potential of progenitors pre- and post-sublineage commitment.

**Fig. 4:**
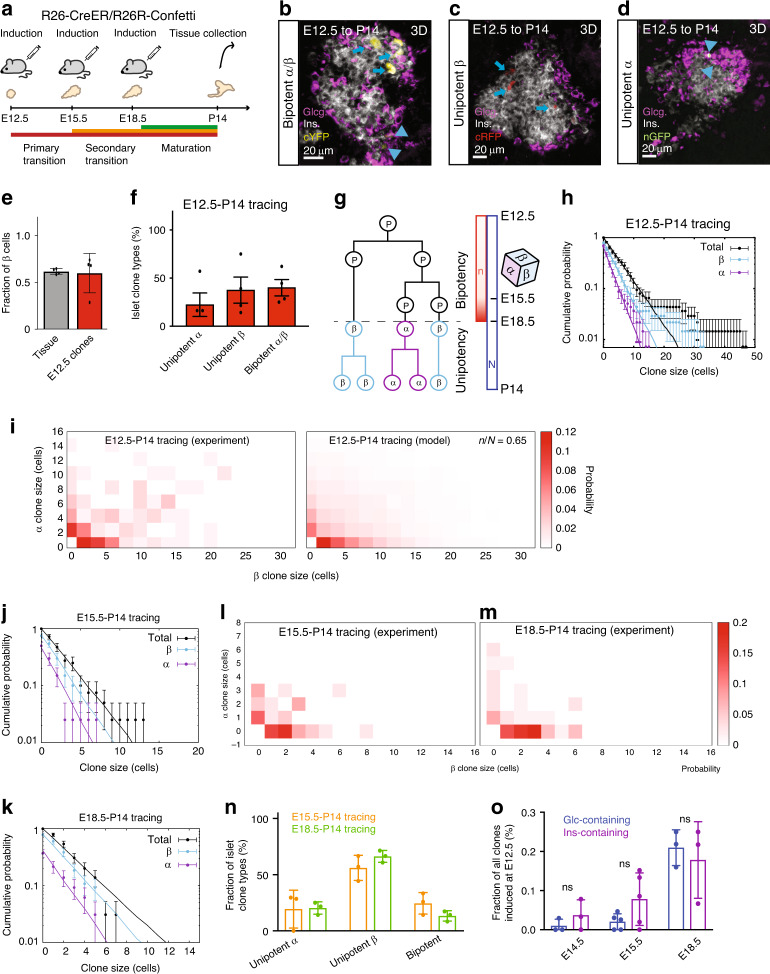
Islet cells originate from bipotent islet progenitors that become sublineage-restricted. **a** Experimental schedule. Representative images from 4 experiments of **b** bipotent, **c** unipotent β-cell, and **d** unipotent α-cell clone (based on >60, 45, >20 recorded images, respectively). Insulin is grey, glucagon is pink. Arrows (arrowheads) indicate insulin+ (glucagon+) cells. **e** Fraction of β-cells (normalised to α+β-cell number) at P14 in islets (*n* = 36 islets from *N* = 4 mice) and clones traced from E12.5 to P14 (*n* = 138 clones from *N* = 4 mice; mouse with low ratio contained only 7 clones), quantified volumetrically from 100 μm section. Error bars show average ± SD. **f** Percentage of clone types (*n* = 138 clones from *N* = 4 mice). Error bars show average ± SEM. **g** Typical model lineage (“Methods”): following endocrine commitment, progenitors undergo average of *n* rounds of stochastic division after which they become sublineage-restricted choosing randomly between α- or β-cell fate in ratio 1:2 (cf. “loaded dice”). Between E12.5 and P14, islet progenitors and progeny undergo average of *N* rounds of division. For simplicity, model excludes differentiation into minority sublineages. **h** Cumulative size distribution of islet clones traced from E12.5 to P14 disaggregated by composition (total black, α-cell purple and β-cell blue). Data shown as dots (*n* = 138 clones from *N* = 4 mice, error bars show average ± SD) and fitted model predictions as lines. **i** Joint distribution of α- and β-cell numbers in islet clones from E12.5 to P14 tracings from data (left) and model (right) (main text and Methods). Cumulative size distribution of islet clones (key as in **h**) traced from **j** E15.5 to P14 (*n* = 40 clones from *N* = 3 mice) and **k** E18.5 to P14 (*n* = 65 clones from *N* = 3 mice). Joint distribution of α- vs. β-cell numbers in clones traced from **l** E15.5 and **m** E18.5 (cf. model prediction in Supplementary Fig. 5a, b). **n** Percentage of clone types traced from E15.5 to P14 (*n* = 40 clones from *N* = 3 mice) and E18.5–P14 (*n* = 65 clones from *N* = 3 mice). Error bars show average ± SD. **o** Percentage of clones contributing to insulin+ or glucagon+ lineage from E12.5 to E14.5, E12.5 to E15.5 and E12.5 to E18.5 tracings, co-stained with DBA and glucagon, or DBA and insulin, showing no statistically significant difference for all timepoints (*P* > 0.2, Mann–Whitney). *n* = 105 (104) clones from *N* = 3 mice for E14.5 insulin/DBA (glcg/DBA) (*P* = 0.40, two-tailed Mann–Whitney test). *n* = 140 (137) clones from *N* = 5 mice for E15.5 insulin/DBA (glcg/DBA) (*P* = 0.30, two-tailed Mann–Whitney test). *n* = 58 (65) clones from *N* = 3 mice for E18.5 insulin/DBA (glcg/DBA) (*P* = 0.80, two-tailed Mann–Whitney test). Error bars show average ± SD. Source data provided as Source Data file.

To map the potency of islet progenitors, we performed additional lineage tracings at multiple timepoints (E12.5–P14, 138 clones from *n* = 4 mice; E15.5–P14, 33 clones from *n* = 3 mice; and E18.5–P14, 60 clones from *n* = 3 mice) using the *R26-CreERT2/R26R-Confetti* mouse model (Fig. 4a). As with the E12.5 tracing (Fig. 2h), the islet clone size distributions from later induction times followed an exponential dependence, consistent with stochastic cell division (“Methods”). To define the composition of islet clones, we combined staining for glucagon, insulin and the nuclear marker, DAPI (Fig. 4b–d, Supplementary Fig. 2 and “Methods”). Cells negative for both insulin and glucagon were identified, but at such low abundance (<3% in all tracings) that statistically meaningful quantitative fate mapping was unfeasible. Importantly, for all tracings, we found that the ratio of α- to β-cells within clones at P14 matched the global ratio within islets, as assessed by volumetric analysis (estimated at around 1:2, Fig. 4e, Supplementary Fig. 3a and “Methods”), confirming the representativeness of the induced population. (Note that the proportion of β-cells within islet clones continued to increase during the later stages of postnatal growth.) Since, at the E12.5 induction time, 48% of islet cell-containing clones were bipotent, containing both α- and β-cells (Fig. 4f), it followed that sublineage restriction is not fully resolved by this stage. (Note that, here, and in the remainder of this section, we reserve the term bipotent to refer to the presence of both α- and β-cell sublineages in individual clones. Moreover, proliferative cells that are restricted to either the α- or β-cell sublineages are referred to as “precursors”, reserving “progenitor” for cells that are bipotent, capable of contributing both ductal and islet cells, or α- and β-cells.)

To interpret the compositional data, we first reasoned that, if the higher proportions of β-cells were due to enhanced proliferation of β-cell precursors, then unipotent β-cell clones should be larger than unipotent α-cell clones. However, both showed the same average size (Supplementary Fig. 3b), arguing against differences in the division rate between sublineages. Instead, this finding suggested that the bias towards β-cell fate arises from more frequent commitment of progenitors into the β-cell sublineage, with β-cell precursors having the same subsequent rate of proliferation as α-cell precursors. We note that differences in division rate would also be manifest in other signatures, such as non-exponential clone size dependencies (see “Methods” Alternative models and parameter-fitting and Supplementary Fig. 3). In further support of this conclusion, we found that unipotent β-cell clones were around twice as abundant as α-cell clones on average (Fig. 4f) across all tracings, confirming that the bias in islet composition reflects a persistent differentiation bias of bipotent progenitors towards the β-cell lineage.

We then questioned the relative timing of α/β-cell fate acquisition, turning to a modelling-based approach to analyse the data. Specifically, we proposed that fate restriction into the two main sublineages (α- and β-cell) occurs after *n* rounds of division following the initial commitment of progenitors into the endocrine lineage, with the particular sublineage allocated stochastically according to the measured α- to β-cell ratio (Fig. 4e). (Note that a small degree of cell-to-cell variability in *n* around the average would not change results significantly.) Based on this minimal model (see Fig. 4g for a typical simulation), we sought to determine *n* reasoning that, if *n* were small, meaning that sublineage restriction occurred soon after endocrine commitment, one would observe a predominance of unipotent clones, while if *n* were comparable to *N*, the total number of divisions over the time course, one would observe a high degree of bipotency (Supplementary Fig. 3c). Based on this model, from the average total size of islet clones traced between E12.5 and P14, we inferred some *N* = 2.6 divisions. Then, from a fit to the measured frequencies of unipotent and bipotent clones (Fig. 4f), we obtained an estimate of *n* = 1.7 ± 0.4 divisions (Supplementary Fig. 3c and “Methods”: Alternative models and parameter-fitting).

Based on these estimates, we found that the model could predict quantitatively the exponential dependence of the marginal distribution of islet clone size (Fig. 4h), i.e. the distribution of α-cell clone size independent of β-cell number and vice versa. Furthermore, the model predicted correctly that the global α- to β-cell ratio seen in the overall tissue should be observed both in the respective unipotent clone numbers, and in the ratio of α- to β-cells within bipotent clones (Supplementary Fig. 3a). Incorporating small levels of apoptosis in the model did not give rise to markedly different joint clone size distributions (Supplementary Fig. 3l), while based on Caspase 3 expression, apoptosis in pancreas appeared to be minimal at E14.5, E15.5 and E18.5 (Supplementary Fig. 3n–p). Finally, we found that the model could predict accurately the joint distribution of α- and β-cell clone size (Fig. 4i).

As defined, the model predicts only the effective timing of fate transitions based on the number of rounds of division following commitment to the endocrine lineage. Based on average clone sizes from the E12.5, E15.5 and E18.5 tracings (Fig. 4h–k, Supplementary Figs. 4 and 5a–d), conversion of this estimate into a time-scale suggests that the bulk of commitment of bipotent progenitors from the E12.5 induction occurs around E15 and lasts until E18 (“Methods”, Alternative models and parameter-fitting). Consistent with this reasoning, a detailed fate mapping over these time windows (Fig. 4j–n, Supplementary Figs. 4a–f and 5a–i) confirmed a drastic reduction in the frequency of bipotent islet clones from the E15.5–P14 tracing (18%), with this fraction becoming even smaller in the E18.5–P14 tracing (<10%) (Fig. 4f, n). Importantly, using parameters obtained from the modelling of the E12.5–P14 data, we found that the model provided a consistently good fit across the range of metrics above at all timepoints (Fig. 4j–m and Supplementary Fig. 5a–e, g, h).

These results were supported by additional short-term tracings, which showed that few clones traced between E12.5 and E14.5 had already up-regulated markers of endocrine fate, with a gradual increase of insulin+ or glucagon+ cells in clones traced from E12.5 to E15.5, and E12.5 to E18.5 (Fig. 4o and Supplementary Fig. 2l–r). Concentrating on clones containing primitive ductal epithelium (PDE) and/or islet components, E14.5 clones were overwhelmingly of PDE origin, with a gradual increase in the frequency of unipotent islet clones and PDE–islet clones at E15.5 and E18.5 (Supplementary Fig. 2l–r), consistent with the progressive specification of PDE cells into either of the islet sublineages during PDE clone expansion. From the analysis of clones traced from E12.5 to E14.5, E12.5 to E15.5 and E12.5 to E18.5, combined with insulin/DBA and glucagon/DBA immunostaining, we did not observe insulin+ or glucagon+ cell-contributing clones in PDE or ducts (Supplementary Fig. 2l–r), although PDE localisation and endocrine lineage segregation might be temporally correlated^33^).

To further study the spatial location of early endocrine progenitors and their progenies, we stained embryonic tissue for chromogranin A (Chrom A). While at E15.5 Chrom A+ cell clusters were small and did not present a typical islet shape (Supplementary Fig. 2g–k), we found that at E18.5 Chrom A+ areas showed a wide range of sizes, with some areas presenting fully formed islet morphology, while others appeared as very small clusters (Supplementary Fig. 2g, h). We then turned to the E12.5–E18.5 tracing. Within the more fully formed islet clusters, we observed that clones tended to be more fragmented (Supplementary Fig. 2i), while smaller Chrom A+ clusters were associated with more cohesive clones at the interface of DBA+ and Chrom A+ cells. Notably, although many cells within clones were only Chrom. A+ or only DBA+, some were DBA+ Chrom. A+, suggesting that specification into the endocrine lineage may occur already in the PDE (Supplementary Fig. 2j, k).

Finally, the ratio of α- to β-cells is known to change during postnatal growth in a manner consistent with preferential β-cell proliferation^25^. To confirm this prediction, we performed additional tracings from E12.5 to P28 (Supplementary Fig. 5j–o). The frequency of bipotent islet clones stayed constant compared to the E12.5–P14 tracing (Supplementary Fig. 5l), consistent with unipotency during postnatal growth. We found that, in contrast to the E12.5–P14 tracings, the average β-cell clone size increased by a factor 2, whereas the α-cell clone size did not change, consistent with the observed tissue ratios (Supplementary Fig. 5k–o). This argued that the expansion of the β-cell compartment post-P14 is driven by enhanced β-cell renewal, which comprises a single equipotent population of precursors, as evidenced again by the exponential dependence of the clone size distribution (Supplementary Fig. 5m).

Importantly, in contradiction with existing hypotheses^14,20,32^, our findings showed that a rigid temporal sequence of islet cell sublineage restriction (α-cell production occurring substantially in advance of β-cell production) was not compatible with the data: If restriction to the α-cell lineage occurred early in development (around E10) while β-cells emerged after E13, one would predict almost pure unipotency of islet clones, a behaviour ruled out by the data (cf. Fig. 4f and Supplementary Fig. 3d, e, g). However, we could not rule out a modest temporal delay (on the timescale of the cell cycle) in the appearance of different sublineages, even at the later stages of embryonic development, leaving open the possibility that local egression of α-cell restricted precursors from ducts might precede that of β-cell restricted precursors^17^ (see “Discussion”). However, from short-term tracings, E12.5–E14.5, E12.5–E15.5 and E12.5–E18.5, clones analysed at E14.5, E15.5 and E18.5 showed no statistically significant difference in the contribution to insulin+ and glucagon+ cells (Fig. 4o, *P* > 0.2 at all timepoints, Mann–Whitney).

### Lineage tracing of *Ngn3*-expressing progenitors

**Fig. 5:**
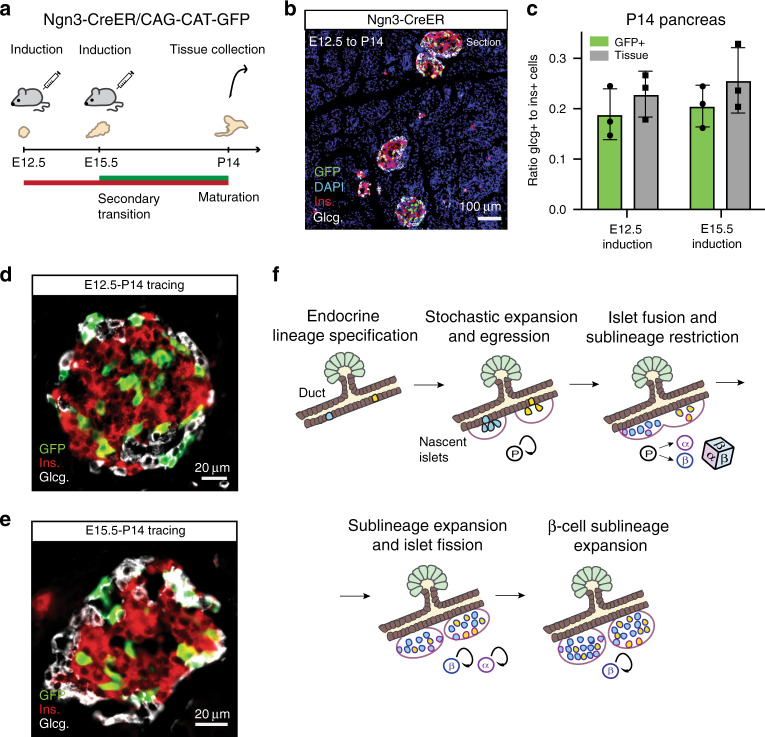
Lineage tracing using Ngn3 promoter confirms contemporary specification of α and β-cell lineages. **a** Experimental schedule for *Ngn3-CreER* lineage tracing at non-clonal levels of induction. **b** Wide-field section of tissue showing typical labelling efficiency based on 3 experiments (and >10 images) from E12.5 to P14 tracing with labelled cells marked by GFP. **c** Quantification of the ratio of glucagon+ to insulin+ cells on thin sections from islets as a whole and from labelled (GFP+) cells from E12.5 to P14 and E15.5 to P14 tracings (*n* = 83 and *n* = 81 islets, respectively, from *N* = 3 mice). Error bars show mean ± SD. Representative images of GFP+ cells within islets based on 3 experiments (and >25 images each) traced **d** from E12.5 to P14 and **e** from E15.5 to P14. Insulin is red and glucagon is white for (**b**, **d**, **e**). **f** Summary of the key events in islet formation based on current findings: endocrine progenitor cell commitment, stochastic progenitor expansion and local egression from ducts, islet sublineage restriction and islet fusion, sublineage expansion and islet fission, and continuing β-cell sublineage expansion. Source data provided as Source Data file.

Finally, to further challenge our findings, we turned to a targeted lineage tracing strategy, using a *Ngn3-CreER™;CAG-CAT-EGFP* reporter mouse model to mark *Ngn3*+ cells^34–36^. To track potential changes in fate bias at the tissue level, mice were non-clonally induced at E12.5 or E15.5 and tissue fixed at P14 (Fig. 5a and Supplementary Fig. 4g, h). Consistent with Ngn3 being a marker of endocrine commitment, lineage labelled cells were restricted to islets (Fig. 5b–e). To quantify the composition of labelled cells, thin tissue sections were immunostained for glucagon and insulin to determine the labelled α- and β-cell fractions benchmarked against the composition of the total islet population (Fig. 5b–e). Notably, comparison of labelled cells with the tissue average showed no statistically significant difference at both induction times (Fig. 5c). Although these findings do not support a fate bias during the later stages of embryonic development^30^, they corroborate our findings based on the unbiased *R26-CreERT2*/*R26R-Confetti* tracings that, at these induction times, sublineage restriction into the α- or β-cell sublineage occurs in a largely contemporary manner.

## Discussion

Using a combination of clonal lineage tracing and biophysical modelling, we have traced the cellular dynamics of islet specification during embryonic and early postnatal development in mouse. These results showed that, following commitment to the endocrine lineage, islet progenitors produced at E12.5 or later amplify through an average of 2–3 rounds of symmetric cell division before becoming sublineage restricted, choosing stochastically between α- or β-cell fates in the ratio of around 1:2 (Fig. 5f). Whether stochastic cell fate allocation follows from an intrinsic cell-autonomous programme or is mediated by signals from neighbouring cells remains an important question for future studies.

In the course of islet development, endocrine progenitors egress from ducts forming nascent islets that progressively coalesce into compound polyclonal structures^16^ that become further dispersed during islet growth, before undergoing a low rate of fission during postnatal growth. Such behaviour is in line with the findings of a recent study showing that islet progenitor egression from ducts involves a highly local process^17^. This combination of progenitor coalescence, stochastic amplification and subsequent islet fission, leads to the emergence of an exponential-like distribution of islet sizes. Interestingly, a similar exponential-like distribution is found in human with comparable mean islet size, suggesting that the mechanism of size regulation may be conserved^18^.

Previously, it has been hypothesised that endocrine cell generation occurs in a step-wise, although partially overlapping, fashion^14,17,20,32^. However, consistent with the results of unbiased lineage tracing, our targeted lineage tracing studies based on the *Ngn3* promoter (both from E12.5 and E15.5) showed no evidence of strong temporal sequencing in the respective allocation of α- and β-cell fate after ~E13.5. Instead, quantitative analysis of the clonal fate data suggested that, following an early amplification phase, commitment to the α- and β-cell sublineages occurs stochastically in a near-contemporary manner, with probabilities consistent with the abundance of cell sub-types. Notably, the use of an unbiased lineage tracing strategy (based on the *R26* promoter) enables the induction of cells of both high and low *Ngn3* levels, unlike the previous lineage tracing strategies that rely on *Ngn3* promoter activity^10^. Importantly, while highlighting an initial phase of α-cell specification until ~E14.5, recent studies suggest a subsequent phase of simultaneous α- and β-cell generation, with cells derived from a common bipotent progenitor^17,37,38^.

Importantly, a recent study probing this question via single-cell RNA-sequencing between E13.5 and E15.5 demonstrated that, although cells expressing α-cell differentiation markers start to appear around 0.5–1 day earlier than β-cell markers, both cell types continue to be produced in an exponentially increasing manner over the next few days, quadrupling each day^17^ between E13.5 and E15.5. Such behaviour is consistent with our analysis as, in an exponential growth regime, the bulk of islet cells would be produced at the latest stage, effectively dwarfing any small biases present earlier in development. Whether the hallmark “mantle-core” structure observed in islets^7,17,33^ develops through the sorting of randomly allocated α- and β-cells (which is consistent with the extensive clonal fragmentation we observed in islets), or whether α-cell restricted precursors egress from ducts slightly in advance of β-cell restricted precursors is left as a question for future studies. In summary, our results provide insight into the functional dynamics of islet fate allocation, providing a quantitative platform to study the molecular programmes that effect the timing of sublineage restriction and the statistics of individual cell fate choice.

## Methods

### Experimental model

For the unbiased lineage tracing, *R26*-CreERT2 and *R26R*-Confetti mice (JAX STOCK Gt(ROSA)26Sortm1(CAG-Brainbow2.1)Cle/J)^39,40^, were crossed. For lineage tracing of embryonic *Ngn3*-positive islet progenitor cells, *Ngn3-CreER*^*TM*^^34^ males were crossed with *CAG-CAT*-EGFP^36^ females. All mice were of *C57BL/6J* background.

Animals of mixed gender were used; littermates were housed together and tissues were collected before weaning. Mice were kept at ambient temperature of 19–23 °C, humidity of 45–65%, and 12 h light/12 h dark cycle. This research has been regulated under the Animals (Scientific Procedures) Act 1986 Amendment Regulations 2012 following ethical review by the University of Cambridge Animal Welfare and Ethical Review Body (AWERB) for *R26*-CreERT2 lineage tracing; and the approval of the Institutional Animal Care and Use Committee of National Institutes of Natural Sciences, Japan, for *Ngn3-CreER*^*TM*^ lineage tracing.

### Induction of lineage tracing

Tamoxifen (Sigma, T5648-1G) was prepared at 10 mg/ml in corn oil (Sigma, C8267) for *R26*-CreERT2 inductions; mice were intraperitoneally injected with Tamoxifen at 0.020 and 0.025 mg per gram of pregnant female for the E9.5 and E12.5 tracings, respectively, and 0.01 mg for E15.5 and E18.5 induction. Pancreas from E14.5, E15.5, E18.5. P14 or P28 neonatal pups was collected. Part of our current data set is based on tissue harvested in an earlier study^6^, but not detailed or analysed there or elsewhere. For *Ngn3*-CreER^TM^ tracing, 4.0 mg of Tamoxifen (Toronto Research Chemicals), solubilized in 100 μl Peanut oil (Sigma-Aldrich) was administered orally into pregnant females once at E10, E12.5 or E15.5. Pups were delivered by Caesarean section at E19.5 (considered as P0) and fed by foster mother. Pancreas from P14 pups were harvested for analysis. For both lineage tracings, pups from mothers without TAM administration were used as a control (ref. ^6^ and Supplementary Fig. 4a, b), whereby male and females of identical genotype as the experimental one were mated. Noon of the day of plug was regarded as E0.5.

### Tissue preparation

Embryonic and neonatal pancreas was fixed in 4% Paraformaldehyde from 45 min to overnight, dependent on its developmental stage, and then washed in phosphate-buffered saline (PBS) extensively. For cryostat sectioning, samples were incubated in 30% sucrose overnight, mounted in OCT and subsequently 100 μm cryostat sectioned as in Figs. 1–4, or 7 μm cryostat sectioned as in Fig. 5.

### Tissue staining

Thick 100 μm cryostat sections were rehydrated in PBS. Sections and whole mount pancreata were blocked overnight in PBS, 2% donkey serum and 0.5% Triton-100X. The samples were incubated in primary antibodies (Chromogranin A, 1:180 from Abcam, ab15160; insulin, 1:100, from Abcam, ab7842; glucagon 1:200, from Abcam, ab10988); and Dolichos biflorus agglutinin (DBA) biotinylated (1:270, from Vectorlabs, B-1035) for 3 days at 4 °C. Secondary antibodies were applied (from Thermo Fisher Scientific) and AF647-Streptavidin (1:800, from Thermo Fisher Scientific, S21374), BV510-Streptavidin (1:600, from Biolegend, 405233) for 2 days at 4°C. All tissues were cleared with RapiClear 1.52 (from SunJin Lab, RC152001).

Thin sections were incubated in 2% Serum, 0.1% Triton-100X in PBS for 30 min, subsequently incubated in primary antibody in 0.01% Triton-100X in PBS overnight (insulin 1:100, from Abcam, ab7842, glucagon 1:200 from Abcam, ab10988, cleaved caspase 3, 1:400, from Cell Signalling, 9664S), washed 3 times for 10 min the next day, and incubated with secondary antibody in 0.01% Triton-100X for 3 h, washed and mounted.

### Confocal microscopy and image analysis

Images of thick section pancreas were acquired using a Leica TCS SP5 confocal microscope for *R26-CreERT2/R26R-Confetti* analysis of Chromogranin A and DBA staining, and for the insulin glucagon analysis of *Ngn3-CreER*^*TM*^*; CAG-CAT-EGFP*. For the analysis of insulin and glucagon staining of *R26-CreERT2/R26R-Confetti* samples, a Zeiss 880 microscope, with the spectral imaging module, was used. For both microscope models, the tiling modules were applied to record the large-scale events. To obtain 3D reconstructed images from Z stacks from an image captured by the Leica SP5 microscope, Imaris software (v8, Bitplane) was used, and numbers of insulin+ and glucagon+ cells were counted in the slice mode. Volumes of objects such as ducts and islets were recorded with Volocity (6.5) software. We estimated the average volume of a cell to be around 300 μm^3^ based on the measurement of single cell clones. By setting intensity thresholds manually for every image, clusters of different confetti colour were identified, and the required parameters computed by the software. Information about the constituent cell types within clusters was collected by analysing co-localisation of clusters with a pancreatic marker in the Z-layer mode of Volocity.

### Cell fate assessment

All analyses were performed on high resolution images. Fate assignment was based on DAPI, DBA, Chromogranin A insulin and glucagon staining. For the assessment of clonality with statistical confidence, the four-colour confetti reporter system was used in the *R26-CreERT2/R26R-Confetti* analysis. As a result, it was necessary to image Chromogranin A and DBA in the same colour channel. However, the respective cell types could be distinguished reliably by morphology and distinct intensity of the staining^6^. Ductal structures were reconstructed using defined threshold of staining intensity.

### Quantification and statistical analysis—statistical inference of clonality

To test for clonality in the tracing data, we first counted all unlabelled islets across the ensemble of pancreatic thick sections, as well as the number of confetti colours in the islets that were labelled. We found consistently that, both for the E9.5 and E12.5 tracings, the majority of islets were fully unlabelled, while the large majority of labelled islets contained marked cells bearing only a single confetti colour (Supplementary Fig. 1h). These results were consistent with a pattern of stochastic and infrequent cell induction. To be confident in the degree of clonality of labelled cell clusters in islets, it was necessary to test statistically that the observed bipotency of islet sublineages was not artificial, arising from the chance induction of two independent unipotent clones of the same confetti colour ending up in the same islet, and being mis-assigned as bipotent. In the simplest null model, where all clones were derived from unipotent progenitors (in the ratio 1/3 α-cells and 2/3 β-cells), and the colour distribution were 25% GFP, 25% RFP, 25% CFP and 25% YFP, there would be four scenarios in which a GFP+ clone could be in the same islet as another clone: In three cases, this would be with a clone of another confetti colour, and in one case it would be with another GFP+ clone. Thus, the probability of two GFP+ clones being found in the same labelled islet (and therefore not distinguishable) is one-third of the probability of finding a GFP+ clone and another differently coloured clone to be in the same islet. In our case, the latter is found to be around p_2col_ = 25% at E12.5. Moreover, in the unipotent null model, these two GFP+ clones have three possible compositions: Either they are both derived from unipotent α-cell clones (prob. 1/9), both from unipotent β-cell clones (prob. 4/9) or arising from different sublineages (prob. 4/9). Thus, the probability of “fake bipotency” would be 4/9*1/3*p_2col (i.e. around 4%). Modifying this simple argument to take into account the observed mosaicism (11% GFP, 39% RFP, 4% CFP and 46% YFP at E9.5; 17% GFP, 38% RFP, 17% CFP and 28% YFP at E12.5), one can estimate numerically the probability for double-labelling to occur in a given islet of cells bearing the same confetti colour. Although this probability is then colour-dependent (and highest for RFP/YFP), numerical simulations of stochastic induction according to the balance of colour probability above allowed us to infer that 91% of islet clusters bearing a common colour on average should be of clonal in origin (with different colours having different frequencies, and thus different threshold of confidence in clonality). From this data, we could thus estimate with high precision the average fraction of a given islet labelled by a clone (and thus the effective number of progenitors that found a given islet, see Fig. 2n).

When performing quantitative analysis of the lineage tracing data, we also kept track of adjacent islets labelled by the same confetti colour (which could be safely estimated as clonal given the low-labelling density). Importantly, the frequency of such clones spanning different islets was also small (8 out of 55) from the E12.5 tracings, consistent with a relatively low fission rate between E12.5 and P14.

To summarise, the initial E12.5 tracing data (Figs. 1 and 2) thus consisted of 69 clonal fragments in isolated islets (from which we extract 69 islet size to clone cluster size ratios, Fig. 2k, l), localised in 51 islets (with some islets containing more than one colour). We obtained 55 reconstructed islet clones when grouping clonal fragments in nearby islets, which we then correlated with the ductal compartment size. In the E9.5 tracing, clones were found to span neighbouring islets more frequently (18 out of 48), consistent with the earlier labelling of multipotent ductal/islet progenitors seeding independently several nearby islets. In total, the initial E9.5 dataset (Figs. 1 and 2) thus consisted of 93 clonal fragments, localised in 73 islets, and we obtained 48 reconstructed clones when grouping clonal fragments in nearby islets.

To assess the potency of islets clones, tracings were repeated and tissue stained for insulin and glucagon expression. To assess whether islet doublets were associated with fission or fusion events, we measured the localisation of clones of a given colour within a doublet, reasoning that fission should result in only a single side being labelled, while fusion should result in both sides containing labelled cells. We restricted the analysis to large clones—comprised of more than *n* > 5 cells—in order to assess with high confidence whether an islet doublet containing cells on only one side was not due to fission and stochastic positioning of all cells on one side, which has an estimated probability (1/2)^*n*−1^, where *n* is the cell number. We note that the analysis of fission and fusion relies on the assumption that clones fragment within islets so that their position is chosen at random and that they can be segregated in two sides upon fission. This behaviour was corroborated by detailed analysis of the clonal data, which showed that the 55 clones traced from E12.5 to P14 were partitioned into some 284 fragments, translating to over 5 fragments per clone. Interestingly, plotting the fragment size distribution of islet clones revealed an extremely good fit to a log-normal distribution, as expected from a recent model of stochastic clone fragmentation^41^ (Supplementary Fig. 1m).

Finally, to assess the overall proportion of β-cells as a fraction of total islet cell number (taken as the sum of α- and β-cells), we measured *n* = 36 islets in *N* = 4 mice at P14 (the same time point used for the clonal tracing from E12.5 to P14), and calculated in each islet the total β-cell volume and α + β-cell volume. This led to a fraction of 0.68 of β-cells over the entire datasets. Applying the same analysis to the clonal data we obtained an overall fraction of 0.66. From this value, we concluded in the main text that the ratio of α:β cells at P14 is approximately 1:2. We also averaged each volume fraction of β-cells over each mouse to display variability as seen in Fig. 4e, Supplementary Figs. 3a and 5f.

### Quantification and statistical analysis—theoretical basis for interpreting lineage tracing data

The theoretical basis of clonal lineage tracing has been studied extensively (see, for example, ref. ^23^). In particular, it has been shown that hallmarks of cell fate behaviour can be found in the statistical behaviour of the clone size distribution. Clones derived from a single equipotent population of progenitors that divide stochastically give rise to a size distribution in which the probability of finding a clone of size *n* is given by the scaling form, $P_n\left( t \right) = \frac{1}{{n\left( t \right)}}f\left( {\frac{n}{{n\left( t \right)}}} \right)$, where *n*(*t*) denotes the average clone size. In the case of stochastic birth or birth-death processes, or in extrinsic neighbour-based loss/replacements in two dimensions or higher, the scaling function has a simple exponential dependence, $f\left( x \right) = \exp \left( { - x} \right)$. As discussed in the main text, the cumulative islet clone size distribution obtained from the E12.5 to P14 tracing also adopts a strikingly exponential size dependence, both when examining the overall clone size, or clone sizes of the separate α- and β-cell compartments. (In line with standards adopted in the literature^42^, we counted *n* > 100 clones from *N* = 4 mice in order to provide enough statistical power to reach this conclusion with statistical confidence. Specifically, exponentiality of the distributions were tested via a Kolmogorov–Smirnoff test with Lilliefors correction, and revealed no statistically significant difference for either of the E12.5 tracings—Fig. 2h and Fig. 4h—even at the *P* = 0.2 confidence threshold.) This is in stark contrast to that found for the E12.5–P14 ductal and acinar clone sizes obtained from the same lineage tracing strategy^6^; these distributions displayed very broad tails, consistent with a model of ductal termini-driven branching morphogenesis based on a branching-annihilating random walk^6,43^. Similarly, we note that the analysis of neocortical development in mice shows a markedly different, Gaussian-like, distribution of clone size (indicative of more deterministic processes), despite average clone sizes being similar to that observed in the current system, arguing that such lineage tracing experiments can distinguish between hypotheses^44^. The collapse of the islet clone size distribution onto an exponential scaling form suggested that islet progenitors function as an equipotent population, driving islet expansion through rounds of stochastic cell division—a simple birth process (see below for detailed parameter fitting). Note that total clone sizes cannot reach zero in these experiments (as this corresponds to complete clonal loss, which is obviously not detectable in a lineage tracing assay based on the analysis of fixed samples), although unipotent clones contain, by definition, zero cells of one type.

To take into account sublineage segregation within the islet compartment, we considered a minimal extension of the model in which endocrine progenitors transit irreversibly from a bipotent phase (capable of generating both α- and β-cells) to a unipotent phase in which the resulting precursors become irreversibly restricted to either the α- or β-cell sublineages. (For simplicity, we neglected other islet lineages whose abundance was below the threshold to achieve a statistically significant analysis.) More specifically, we considered a model in which progenitors *P*, divide symmetrically,

- *P* → *P* + *P* at rate *λ*(*t*)

and transit stochastically and irreversibly into precursors A or B committed to the α- or β-cell sublineages, respectively,

- *P* → *A* at rate *k*_*A*_(*t*)
- *P* → *B* at rate *k*_*B*_(*t*)

Each unipotent precursor *A* and *B* is also able to duplicate at rates *λ*_*A*_(*t*) and *λ*_*B*_(*t*), respectively, which could in principle be different from *λ*(*t*). Further, all of these rates could, in principle, vary over time. For instance, the prevailing model of sequential α and β-cell production would translate to a rate *k*_*A*_(*t*) rapidly increasing during early pancreatic development (E9.5–E12.5), while *k*_*B*_(*t*) only starts increasing from zero later on in development (E12.5–E15.5). It has also been shown that the ductal plexus provides a niche for islet cell production until late embryogenesis; so one may expect that the phase of *P* cell production may also be long-lived, with proliferation (at rate *λ*(*t*)) being overtaken by differentiation into A and B precursors taking place only late in embryogenesis. To circumvent such uncertainties, in fitting the clonal data, we refer to the effective number of divisions performed between different points of the lineage tracing, without having to reference the timings of proliferative expansion.

### Quantification and statistical analysis—alternative models and parameter-fitting

To fit the model to the experimental data, we constructed distributions of clone size and composition numerically using stochastic simulations of at least 10,000 clones, each derived from a single bipotent progenitor cell labelled at the initial time point. We made use of standard bootstrapping methods on the lineage tracing data to build standard deviations, as well as 95% confidence intervals for our predictions. To constrain and test the model, we focussed on the E12.5–P14 *R26-CreERT2*/*R26R-Confetti* tracings, for which the largest data set was available, and where islet clones were resolved with high statistical confidence. However, to further challenge the model, we used the fits to predict clone size and compositional dependences using the E15.5–P14 and E18.5–P14 *R26-CreERT2/R26R-Confetti* tracings, as well as the population-level *Ngn3-CreER*^*TM*^ tracings. To further constrain the model, we made use of several robust features of the data:

First, we noted that the overall α- and β-cell distributions were well-described by a simple exponential size dependence (Fig. 4h, j, k and Supplementary Fig. 5m) (as was the overall clone size distribution), and that α- and β-cell unipotent clones also had similar sizes (Supplementary Fig. 5k). This is not what one would expect in models where the division rate of β-cells would be much larger than α-cells, suggesting that *λ*_*A*_(*t*) ≈ *λ*_*B*_(*t*). Simulating very different proliferation rates for α- and β-cells produced a marked departure from exponentiality (see Supplementary Fig. 3k, where *n*/*N* = 0.333 and *λ*_*A*_ = 4*λ*_*b*_). Indeed, the higher abundance of β-cells over α-cells overall could be explained by the production of a larger number of unipotent β-cell clones as compared to unipotent α-cell clones, and larger numbers of β-cells in bipotent clones (Fig. 4f and Supplementary Fig. 3a, b). This situation prevailed in all tracings, confirming that differences in α- and β-cell production was not due to temporal differences in differentiation rates. This argues that that the rate of transition into the β-cell sublineage is larger than into the α-cell sublineage, *k*_*B*_ > *k*_*A*_. This observation was further confirmed by the E12.5–P28 tracing (Supplementary Fig. 5j–o), where the phenomenology was reversed: α-cell clones had not increased in size since P14, while β-cell clones had more than doubled in size, resulting in exponential distributions with different slopes. This was indicative of a difference in the division rates, *λ*_*A*_(*t*) < *λ*_*B*_(*t*), appearing between P14 and P28, i.e. that the expansion post-P14 of the β-cell compartment becomes driven by preferential proliferation, and not preferential differentiation of bipotent progenitors P into β-cell precursors B, as earlier on. Here, we note that we are able only to refer to effective proliferation rates: If, for example, there were an undisclosed residual base-line rate of cell apoptosis, numerical simulations shows that the join clone size distribution would be largely unaffected (Supplementary Fig. 3l), while the true proliferation rate would be proportionately higher than the inferred effective rate to accommodate the apoptosis rate.

Second, as time is counted here in terms of numbers of divisions, the relative division rate of the bipotent progenitor population is irrelevant and, for simplicity, can be set equal to that of unipotent precursors, and taken as constant: *λ* ≈ *λ*_*A*_ ≈ *λ*_*B*_ (with the total number of divisions and timing of commitment/differentiation being the only relevant quantity).

Third, as mentioned above, if bipotent progenitors and unipotent precursors coexisted in the long term and had highly different dynamics (for instance in their division rate), one would expect to see more complex clone size distributions, which is ruled out by the simple exponential scaling form for all time points. To show this, given that roughly one half of the clones traced from E12.5 displayed bipotent outcomes, we simulated a scenario in which 50% of induced clones were unipotent precursors (either α- or β-cell lineage in a 1:2 ratio), and 50% were bipotent until the end of the tracing. However, if both populations had the same division rate, a fraction of clones derived from bipotent progenitors would still end up with unipotent output due to chance and small number statistics (for instance, a two-cell clone make derive from the stochastic specification of β-cells), and the overall bipotency fractions would be too low to infer bipotency with confidence. The only solution to remedy this is to assume a much larger proliferation rate of bipotent progenitors (so that their clone size is so large as to make chance unipotent outcomes unlikely), while maintaining the overall average clone size of 5.6 cells. We thus simulated stochastic unipotent precursors undergoing *N* = 0.75 divisions and stochastic bipotent progenitors undergoing *N* = 3.25 divisions. However, this resulted in non-exponential clone size distributions, which despite the higher number of fitting parameters compared to our previous simpler model, worsened the fit in particular for the total clone size (Supplementary Fig. 3m).

Thus, the simplest model, consistent with the qualitative features described above (from E12.5 to P14) is one where (i) division rates are constant, and (ii) a transition takes place from bipotency to unipotency (viz. rates *k*_*A*_(*t*) = *k*_*B*_(*t*) = 0 for *t* < *n*/*N*, and *k*_*A*_(*t*) = *K*_*A*_ and *k*_*B*_(*t*) = *K*_*B*_ = 1 − *K*_*A*_ for *t* > *n*/*N*, with constant probabilities *K*_*A*_ (and therefore *K*_*B*_) chosen to match the proportions of α-cells and β-cells found in the organ overall.

From a more quantitative standpoint, the simplest model thus relies on three key parameters: the relative probability to differentiate into α- vs. β-cell fate, *k*_*A*_, the total number of cell divisions between E12.5 and P14,*N*, and the timing of the transition *n*/*N* (expressed as a fraction of *N*). The first and second parameters can be estimated with high precision directly from the data simply by (i) calculating the total ratio of α to β-cells, which leads to the estimate $K_A = 0.37 = 1 - K_B$, and (ii) using the average clone size of 6.1 ± 0.6 cells to estimate *N* = 2.6 ± 0.1 (mean ± SEM). The third parameter needs to be fit via numerical simulations of the model. To constrain the model with the smallest fraction of data (in order to test for consistency on the remainder of the clonal data), we thus resorted to a simple fit of the fraction of bipotent clones (which increases monotonically with the timing of commitment, *n*/*N*). We performed 1000 iterations of the numerical simulation, each time boot-strapping the experimental dataset (to calculate the confidence intervals on bipotency fraction), and found the best fit for *n*/*N* (each time simulating 10,000 clones). This allowed us to find a best fit and construct a 95% confidence interval with *n*/*N* = 65% ± 15%.

With this best-fit value, several aspects of the data, not used in the fitting procedure, can be predicted quantitatively by the model, including the joint clone size distributions of α and β-cell compartments (Fig. 4i). Features of this two-dimensional distribution include: (i) the exponential-like clone size distributions of α- and β-cell compartments (Fig. 4h); (ii) the relative fraction of unipotent α- and β-cell clones (Fig. 4f); (iii) the fact that α- to β-cell ratio is identical when looking either at bipotent clones only or α/β-cell unipotent clones only (Supplementary Fig. 3a); and iv) the correlation and variance of α vs. β-cell number in a clone (Supplementary Fig. 3d, f), showing that the variance increases with the number of cells in a clone, as expected for a binomial distribution arising from random fate assignment between α- and β-cell fate. However, we note that the E12.5–P14 tracing data contained 6 clones (out of 138), which were manifest outliers compared to the model and the rest of the data. These 6 clones all had an average clone size between 3 and 5 standard deviations away from the mean. To improve visual comparison between joint distributions of α- and β-cells, we thus “zoomed in” (in Fig. 4i, compared to Supplementary Fig. 3d) on smaller clones sizes to concentrate on the bulk of the data. Interestingly, despite their size, these outlier clones were “normal” in their ratio of α- and β-cells (i.e. an approximate ratio of 1:2, Supplementary Fig. 3d). Given that we did not find these clones to correlate with areas of larger clonal induction, we reasoned that they were unlikely to arise from fusion events, and might thus arise due to rare hotspots of proliferation during early embryogenesis. This would be consistent with the fact that such outliers were not present in the E15.5 or E18.5 tracings.

To perform a sensitivity analysis, the predictions were contrasted with a model involving earlier sublineage commitment (i.e. mostly unipotent α- and β-cell clones being present, and thus with highly independent α- vs. β-cell compartment sizes, see Supplementary Fig. 3g), or later sublineage commitment (where α- and β-cell compartment size in a clone are maximally correlated, as both derive from the same bipotent progenitor pool, see Supplementary Fig. 3h). Notably, we found an intermediate level of positive correlation between α- and β-cell numbers in E12.5 clones (slope 0.3 ± 0.04, *R*^2^ = 0.27), in quantitative agreement once again with the predictions of the model (Fig. 4i and Supplementary Fig. 3d, i). Alternative models, such as one where a bipotent islet progenitor self-renews while continuously producing unipotent α- and β-cells with stochastic fate allocation, provided poor fits to the data (Supplementary Fig. 3j), including the average and variance of the number of α-cells in clones with a given number of β-cells (Supplementary Fig. 3d, f)

Although our data ruled out distinct phases of α- and β-cell allocation (a feature confirmed by the *Ngn3-CreER*^*TM*^ tracing), one can, of course, introduce additional refinements of the minimal model incorporating, for instance, a defined time for the fate specification of α- and β-cells, as well as small time delays between the allocation of both. Although these additional parameters only improve marginally the quality of the fits, the data can accommodate, for instance, a phase of α-cell allocation (initiating at around *T*_*A*_ ≈ 50%) and β-cell allocation (starting later at around *T*_*B*_ ≈ 65%). Note that these delays are within the confidence intervals of *n*/*N* from the minimal model, and that the model still requires the late specification of α-cells (50% corresponds to around E15.5–E18.5 in embryonic time from the clone size of the different tracings) compared to past proposals in the literature. Finally, the same model as for the E12.5–P14 tracing could explain faithfully the E12.5–P28 tracing (Supplementary Fig. 5j–o) by simply assuming that α-cells do not proliferate within the later time interval (P14–P28), while β-cells undergo an additional round of division. This is consistent with clone sizes of each compartment (Supplementary Fig. 5k), as well as overall clonal potency (Supplementary Fig. 5l), the α- and β-cell clone size distributions (Supplementary Fig. 5m), and the joint probability distributions (Supplementary Fig. 5n, o).

### Quantification and statistical analysis—number of progenitors per islet

Finally, our model of clonal dynamics allowed us to infer the mechanisms driving the heterogeneity in islet size itself. From a theoretical perspective, part of this heterogeneity could arise from stochasticity in clonal behaviour. However, given that around 30 progenitors at E12.5 contribute to a given islet, such a “clonal” source of heterogeneity would be small (i.e. the variance from adding 30 Poissonian processes of average *n*_*p*_ ≈ 6, resulting in a Gamma distribution of mean 30*n*_*p*_ ≈ 240 and standard deviation $\sqrt {30} \,n_p \approx 32$), compared to the wide, experimentally observed, distribution of islet sizes. We typically found that the variance in islet volume was close to its average, consistent with the wide distributions previously measured in the literature^18^. Although we could find small correlations between islet size and position towards the centre of the pancreas, islet clone size did not correlate with position. Having ruled out other factors, such as enhanced proliferation of all clones in bigger islets, this allowed us to conclude that the bulk of islet size variance must originate instead from the variability in the number of progenitors making up an islet (which depends both on the kinetics of islet aggregation and subsequent fission dynamics). We note however we cannot exclude within the resolution of the data some spatial variations to the model parameters, such as specification timing or islet size, which could cause additional variability in islet size.

### Reporting summary

Further information on research design is available in the Nature Research Reporting Summary linked to this article.

## Supplementary information

Supplementary Information

Reporting Summary

## Data Availability

The authors declare that all data supporting the findings of this study are available within the article and its supplementary information files or from the corresponding authors upon reasonable request. Source data are provided with this paper.

## Source data

Source Data
