# Supplementary material for: Tracing the cellular basis of islet specification in mouse pancreas
Source: Nat Commun. 2020 Oct 7;11:5037. doi: 10.1038/s41467-020-18837-3 (PMC7541446; doi:10.1038/s41467-020-18837-3)
Supplement: Supplementary file 1 — Supplementary Information [file 41467_2020_18837_MOESM1_ESM.pdf]

Supplementary Information (SI)

Tracing the cellular basis of islet specification in mouse pancreas

Sznurkowska et al.

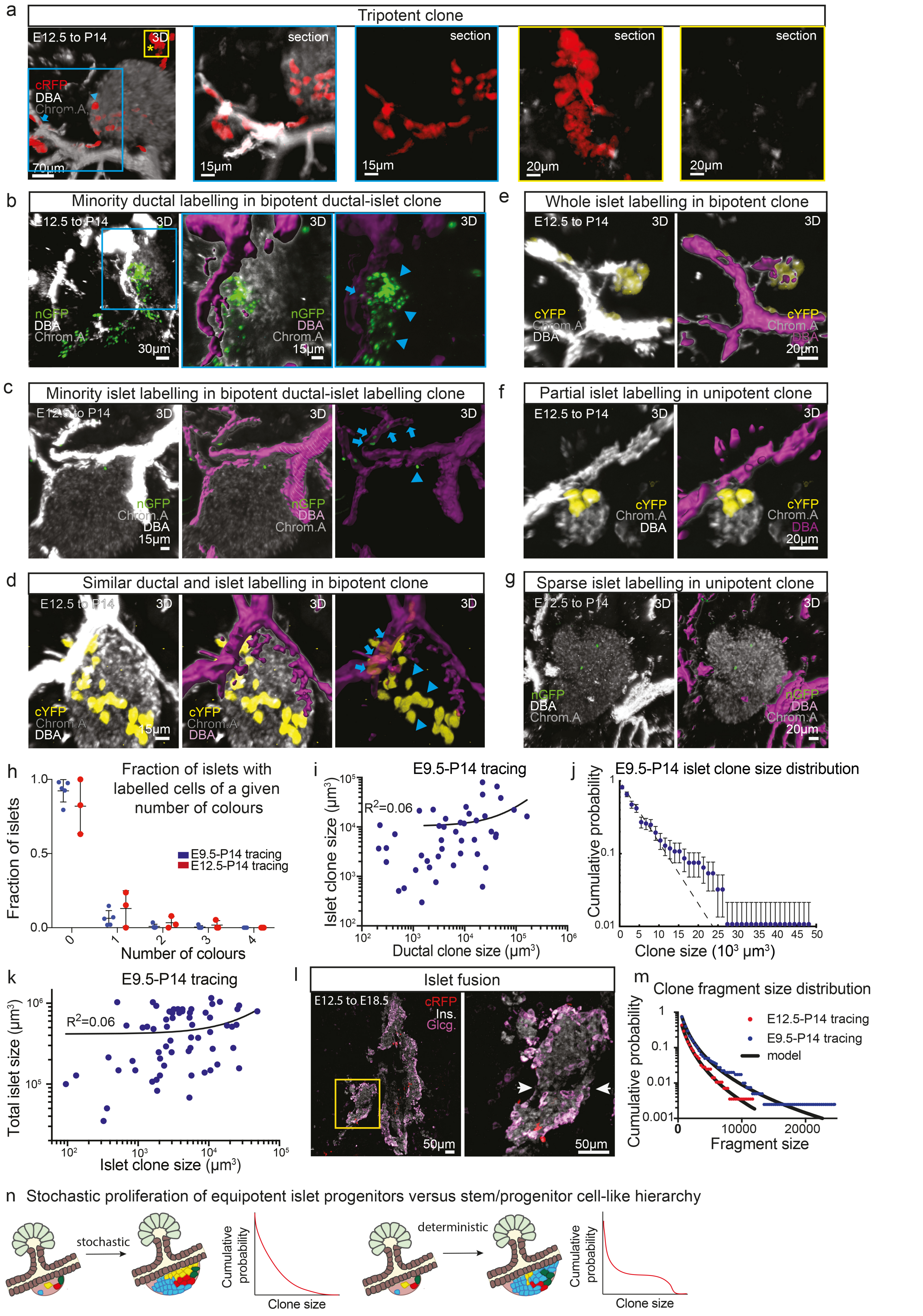

Supplementary Figure 1

Supplementary Figure 1. *Additional images and graphs related to Figures 1-3.*
a-d) 3D projections (left) and ductal reconstruction (middle and right) of islet clones showing: (a) tripotent islet-ductal-acinar clone; different size ratios of islet to ductal component with (b) showing minority ductal labelling, (c) minority islet labelling, and (d) similar ductal and islet labelling in bipotent clones. **e-g**) 3D projections (left) and ductal reconstruction (right) of islet clones displaying different fractions of co-localisation within the islet with (e) whole islet labelling in a bipotent clone, (f) partial islet labelling in a unipotent clone, and (g) sparse islet labelling in a unipotent clone. Purple shows ducts reconstructed based exclusively on intensity of Chromogranin A and DBA, as described in Ref. [6]. Chromogranin A is grey and DBA is white. (a-g) are representative of >20 images from 3 experiments for (a) and >15 images each for (b-g). **h)** Fraction of islets with a given number of colours for E9.5-P14 (n=874 islets from N=5 mice) and E12.5-P14 tracings (n=214 islets from N=3 mice). Error bars show mean±SD. **i)** Relationship between ductal and islet component of multipotent clones. **j)** Cumulative clone size distribution for clone volumes for E9.5-P14 tracing (n=93 clones from N=5 mice). Dashed line shows an exponential fit. Error bars show mean±SD. **k)** Relationship between islet clone volume and islet volume for E9.5-P14 tracings. **l)** Poly-islet structures observed at E18.5 with clones traced between E12.5 and E18.5 (left inset), representative of 15 images from 4 experiments. The right inset shows a zoom on an islet doublet with labelled cells present in only one of the islet domains. White arrows indicate constriction point in the doublet. **m**) Cumulative clone size distribution of islet clone fragments traced from E9.5 (blue) or E12.5 (red) to P14. Points represent data and lines a log-normal fit, as expected theoretically based on random merger and fragmentation of clusters, showing excellent fits at both timepoints. **n**) Schematic representing the stochastic proliferation of equipotent progenitors (left) and the deterministic proliferative output of stem cell-like population (right), with the corresponding cumulative distribution for different clone sizes.

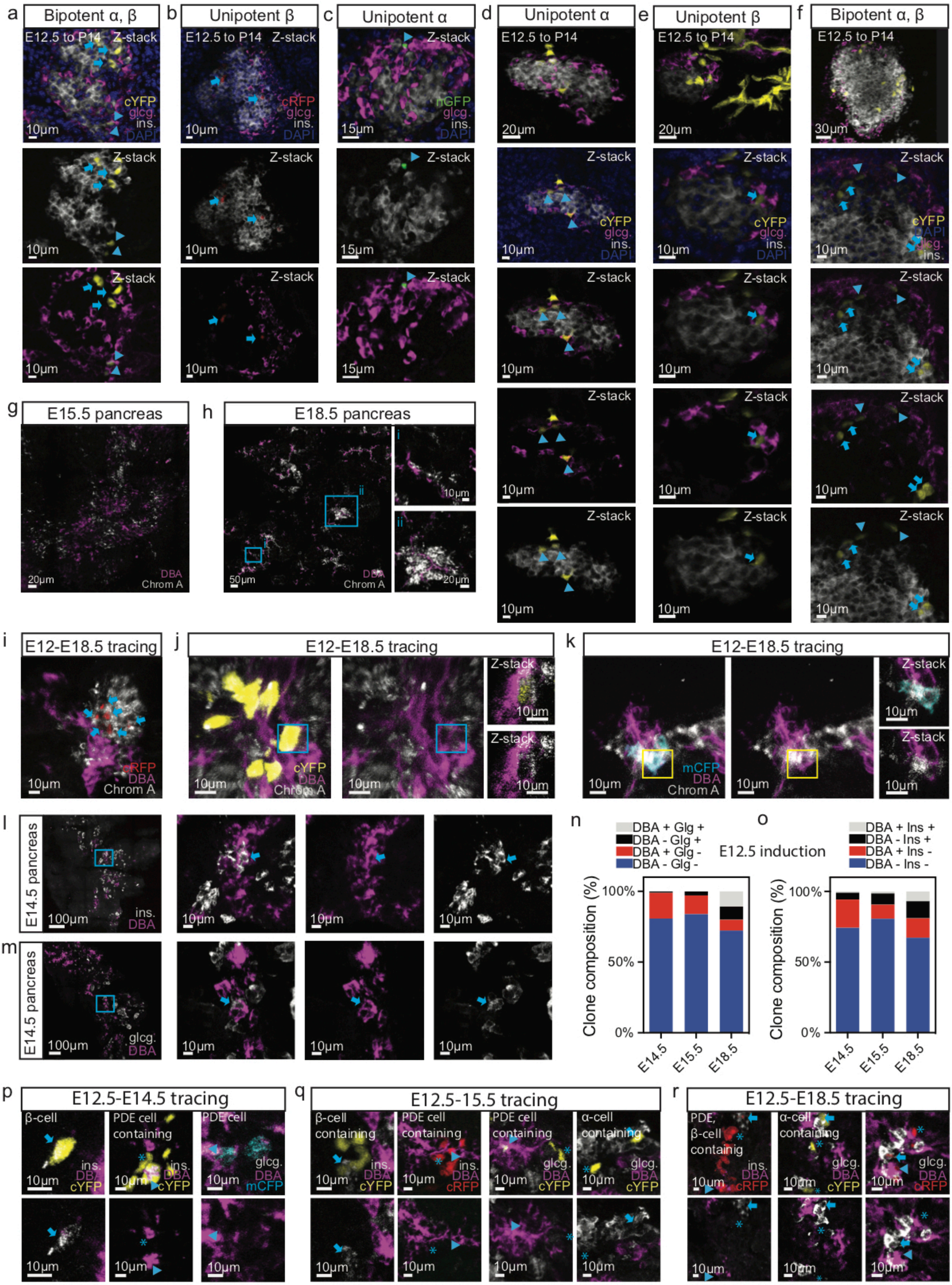

Supplementary Figure 2

Supplementary Figure 2. *Additional images and graphs related to Figure 4.*

a-c) Combinations of channels for sections of 3D images in Fig. 4b-d. Representative clones for E12.5-P14 tracings with 100μm sections co-stained with insulin and glucagon: **d)** α-cell unipotent, **e)** β-cell unipotent, and **f)** α/β-cell bipotent clone. (d-f) are representative of, respectively, >20, 45, >60 images from 4 experiments each. Insulin is grey, glucagon is pink, DAPI is blue. Arrows (arrowheads) indicate insulin+ and (glucagon+) cells. Representative image of chromogranin A and DBA immunostaining of **g**) E15.5 and **h**) E18.5 tissue, based on> 10 images from 4 and 3 experiments, respectively, with latter showing areas with small chromogranin A+ clusters (top-right inset) and more islet-shaped clusters (bottom-right inset). Images of E12.5-E18.5 traced clones representative of 3 experiments (based on >10 images each) showing **i**) a more separated clone and **j,k**) clones clustered at interface of DBA+ and Chromogranin A+ areas with a zoom on cells displaying double-positive characteristics. Representative images of pancreatic tissue at E14.5 immunostained for **l)** insulin and DBA (N=3 mice, representative of >10 images) and **m)** glucagon and DBA (N=3 mice, representative of >10 images), shown as large areas (right) and zooms (left), with occasional small areas with double-positive insulin+ DBA+ and glucagon+ DBA+, respectively (arrow). **n,o)** Outcome of lineage tracing analysis for E12.5-E14.5, E12.5-E15.5 and E12.5-E18.5 tracings (N=3,5,3 mice, respectively), combined with immunostaining of insulin and DBA, or glucagon and DBA, showing progressive increase in fraction of clones contributing to hormone+ lineages between E15.5 and E18.5. Fraction of clones contributing to DBA+ and hormone+ cells (DBA+hormone+), to hormone+ cells only (DBA-hormone+), to ductal cells only (DBA+hormone-), or to neither lineage (DBA-hormone-). Representative images (based on at least 5 images each) for clones traced from E12.5 to **p**) E14.5, **q**) E15.5, and **r**) E18.5 for insulin/DBA and glucagon/DBA analyses. Asterisk indicates non-DBA non-hormone cell, arrowhead indicates DBA+ cell, arrow indicates hormone+ cell. Hormone+ clones, hormone+ cell-containing clones, primitive ductal epithelium (PDE) clones, PDE cell-containing clones, hormone+ and PDE+ cell-containing clones are shown. (N=3 mice, n=105 clones for E14.5 insulin/DBA; N=3 mice, n=104 clones for E14.5 glcg/DBA; N=5 mice, n=140 clones for E15.5 insulin/DBA; N=5 mice, n=137 clones for E15.5 glcg/DBA; N=3 mice, n=58 clones for E18.5 insulin/DBA; N=3 mice, n=65 clones for E14.5 for glcg/DBA).

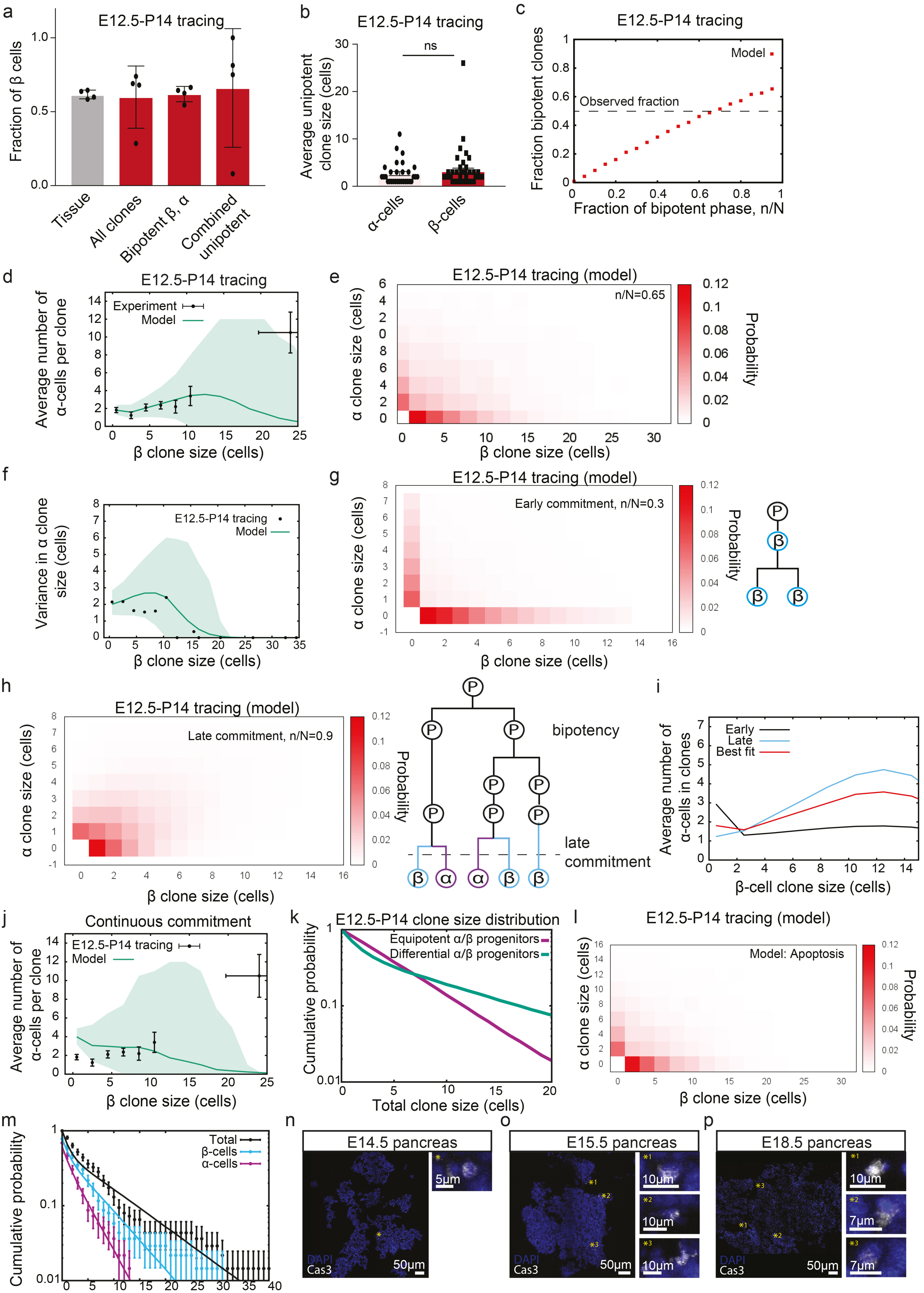

Supplementary Figure 3

Supplementary Figure 3. *Additional graphs and images related to Figure 4.*

a) Fraction of β-cells (normalized to α+β-cells) (n=36 islets from N=4 mice) in all clones traced from E12.5-P14 as well as bipotent α,β-cell clones and combination of unipotent α- or β-cell clones (n=138 clones from N=4 mice; one mouse with few β-cells overall consisted of only 7 clones). Error bars show average±SD. **b)** Size of clones traced from E12.5-P14 showing no statistically significant differences between unipotent α- and β-cell clones (n=30 and n=43 clones from N=4 mice, respectively). Error bars show average±SD. **c)** Predicted (dots) and experimental (dashed) fraction of bipotent clones as a function *n/N* (see main text)**. d)** Average α-cell size shown as function of β-cell size in clones traced from E12.5-P14. Data shown as points (n=138 clones from N=4 mice) and fit as line with shading representing 95% confidence interval. Based on scarcity of clones, the data point for largest β-cell count combines several entries (Methods). Error bars show average±SD. **e**) Predicted joint distribution of α- and β-cell number in islet clones from E12.5-P14 tracings (reproduced from Fig. 4i for comparison with g,h,l). **f)** Experimental (dots) vs. theoretical (line) variance in α-cell size as function of β-cell size in E12.5 clones, supporting model of stochastic α/β-cell fate allocation. Shaded areas represent 95% confidence intervals**.** Sensitivity analysis: Predicted joint distribution of α- vs. β-cell numbers in E12.5 clones for **g)** early or **h)** late commitment. **i)** Corresponding mean α-cell clone size as function of β-cell clone size, compared to best fit (Fig. 4i). **j)** Alternative model with continuous specification of α- and β-cells predicts incorrect correlation between α- and β-cell clone size (n=138 clones from N=4 mice, Methods). Error bars show average±SD. **k**) Cumulative total clone size distribution under null hypothesis (best fit from Fig. 4 where α- and β-cells have same proliferation rate, purple), or under alternative model where β-cells proliferate much faster than α-cells (division rate of β-cells doubled and α-cells halved, n/N=0.333). Shows that exponential distributions only expected in absence of substantial population heterogeneity. **l**) Predicted joint distribution of α- vs. β-cell numbers in E12.5 clones when, on average, one apoptosis event takes place per clone per time course, balanced by increase of average division number to N=3.5, and all other parameters kept constant, showing that results are largely insensitive to small rates of cell loss. **m**) Cumulative clone size distribution (total black, α-cells purple, β-cells blue) in data (n=138 clones from N=4 mice) vs. alternative model (lines) in which 50% of induced clones are unipotent and the rest are bipotent (Methods), showing non-exponential dependencies. Error bars show average±SD. **n-p**) Representative images from 3 experiments each based on >10 recorded images each of tissue immunostained for Caspase 3 at E14.5, E15.5 and E18.5 showing only a few Caspase 3+ cells labelled by asterisk on left and zoom in insets on right. Source data provided as Source Data file.

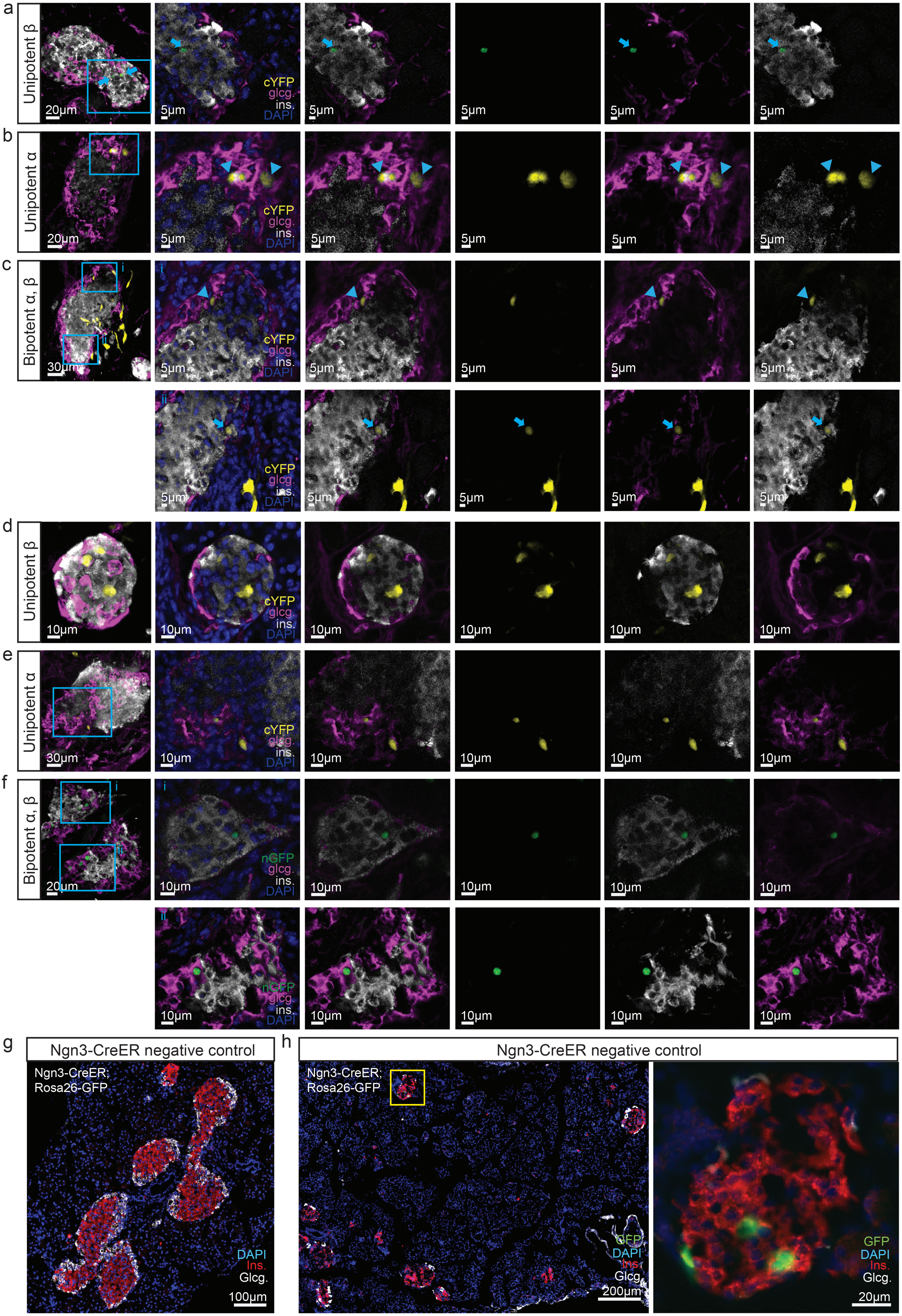

Supplementary Figure 4

Supplementary Figure 4. *Additional images related to Figures 4 and 5.*

Representative clones for the **a-c)** E15.5-P14 and **d-f)** E18.5-P14 *R26-CreERT2/R26R-Confetti* traced clones within thick 100μm sections co-stained with insulin and glucagon presented as 3D projection and zoomed individual Z-stacks: a,d) unipotent insulin+ clones, b,e) unipotent glucagon+ clones, and c,e) bipotent clones. Grey is insulin, pink is glucagon, DAPI is blue. Images representative of at least 10 recorded images from 3 experiments for each tracing. Negative control for *Ngn3*-CreER lineage tracing. **g)** Majority of tissue showed no GFP expression, with **h)** rarely labelled islets (2 labelled islets observed in 3 pancreata). Insulin is red, glucagon is white, DAPI is blue. Representative images based on 30 recorded images from N=3 mice. Labelling efficiency in induced mice is shown in Fig. 5b.

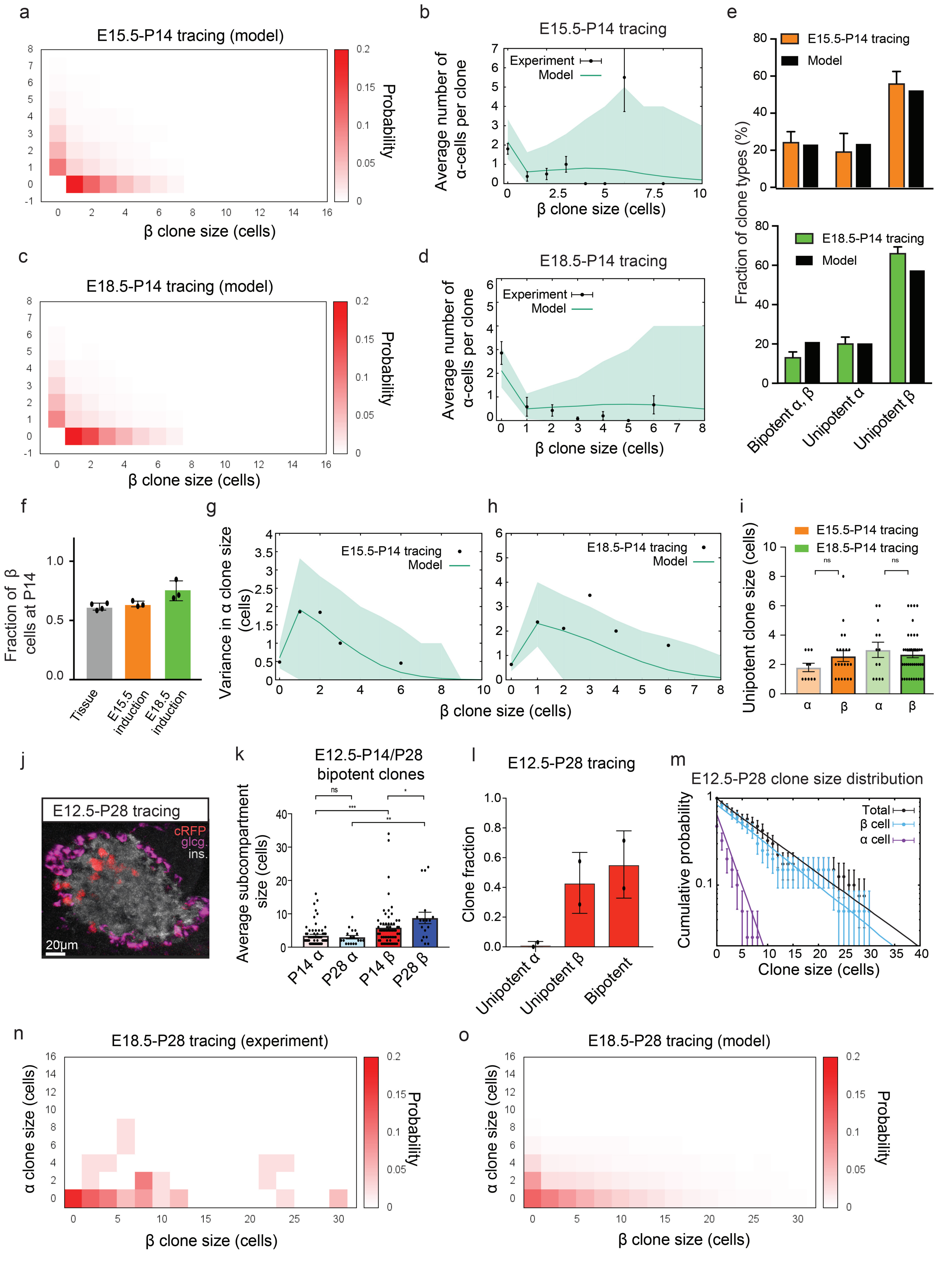

Supplementary Figure 5

Supplementary Figure 5. *Additional images related to Figure 5.*

Predicted joint distribution of α- and β-cell numbers in clones traced from **a)** E15.5-P14 and **b)** E18.5-P14, displaying markedly reduced degree of bipotency compared to E12.5 tracing (Fig. 4), and good agreement with data (Fig. 4j,k). n=40 clones from N=3 mice. Error bars show average±SD. Average α-cell compartment size as a function of β-cell size, showing both data (dots) and theory (lines) for **c)** E15.5 and **d)** E18.5 tracings. Shaded areas represent 95% confidence intervals**.** n=65 clones from N=3 mice. Error bars show average±SD. **e)** Potency of E15.5 (top, orange) and E18.5 (bottom, green) clones compared to model prediction (same parameters as Fig. 4j,k). n=40 clones from N=3 mice for E15.5-P14 tracings, n=65 clones from N=3 mice for E18.5-P14 tracings. Error bars show average±SD. **f)** Fraction of β-cells in P14 islets (n=36 islets from N=4 mice) and islet clones traced from E15.5-P14 and E18.5-P14. n=40 clones from N=3 mice for E15.5-P14 tracings, n=65 clones from N=3 mice for E18.5-P14 tracings. Error bars show average±SD. Experimental (dots) vs. theoretical (line) variance in α-cell compartment size as a function of β-cell size in **g)** E15.5 and **h)** E18.5 clones, showing that model of stochastic α/β-cell fate allocation provides good fit to data. n=40 clones from N=3 mice for E15.5-P14 tracings, n=65 clones from N=3 mice for E18.5-P14 tracings. **i)** Clone sizes for unipotent α- and β-cell clones for E12.5-P14, E15.5-P14, and E18.4-P14 tracings, showing significant differences (P>0.2, two-tailed Mann-Whitney tests). n=40 clones from N=3 mice for E15.5-P14 tracings, n=65 clones from N=3 mice for E18.5-P14 tracings. Error bars show average±SD. **j)** Representative image (based on >30 recorded images) of E12.5-P28 traced clone (N=3 mice), showing expansion of insulin+ cell compartment. Insulin is grey, glucagon is pink. **k)** Size and potency of P28 clones induced at E12.5. Left panel: Mean size for α- and β-cell compartment in bipotent clones at P14 and P28 showing that, while α-cells do not expand in number between P14 and P28 (P=0.88, two-tailed Mann-Whitney test), β-cells expand by a factor of ~2 (P=0.04, two-tailed Mann-Whitney test), leading to significantly different sizes (P=0.001 at P14, P=0.002 at P28, two-tailed Mann-Whitney tests). Error bars show average±SD. **l**) Clone potency at P28, displaying similar degree of bipotency as P14 clones (Fig. 4f). Error bars show average±SD. **m)** Experimental (dots) vs. theoretical (lines) cumulative size distribution of clones induced at E12.5 and traced until P28 (total purple, α-cell cyan, β-cell green). Data is well-fit by an exponential, as expected for stochastic duplications and fate choices. n=33 clones from N=3 mice. Error bars show average±SD. **n,o)** Experimental (left) vs. theoretical (right) joint distribution of α vs. β-cell numbers in clones traced from E12.5-P28. n=33 clones from N=3 mice. Source data provided as Source Data file.
