# Supplementary material for: Tracing the cellular basis of islet specification in mouse pancreas
Source: Nat Commun. 2020 Oct 7;11:5037. doi: 10.1038/s41467-020-18837-3 (PMC7541446; doi:10.1038/s41467-020-18837-3)
Supplement: Supplementary file 2 — Reporting Summary [file 41467_2020_18837_MOESM2_ESM.pdf]

nature research

Corresponding author(s): Benjamin Simons, Anna Philpott

Last updated by author(s): 2020/07/29

Reporting Summary

Nature Research wishes to improve the reproducibility of the work that we publish. This form provides structure for consistency and transparency in reporting. For further information on Nature Research policies, see Authors & Referees and the Editorial Policy Checklist .

Statistics

For all statistical analyses, confirm that the following items are present in the figure legend, table legend, main text, or Methods section.

n/a	Confirmed	
☐	☒	The exact sample size (*n*) for each experimental group/condition, given as a discrete number and unit of measurement
☐	☒	A statement on whether measurements were taken from distinct samples or whether the same sample was measured repeatedly
☐	☒	The statistical test(s) used AND whether they are one- or two-sided<br>*Only common tests should be described solely by name; describe more complex techniques in the Methods section.*
☐	☒	A description of all covariates tested
☐	☒	A description of any assumptions or corrections, such as tests of normality and adjustment for multiple comparisons
☐	☒	A full description of the statistical parameters including central tendency (e.g. means) or other basic estimates (e.g. regression coefficient) AND variation (e.g. standard deviation) or associated estimates of uncertainty (e.g. confidence intervals)
☐	☒	For null hypothesis testing, the test statistic (e.g. *F*, *t*, *r*) with confidence intervals, effect sizes, degrees of freedom and *P* value noted<br>*Give P values as exact values whenever suitable.*
☒	☐	For Bayesian analysis, information on the choice of priors and Markov chain Monte Carlo settings
☒	☐	For hierarchical and complex designs, identification of the appropriate level for tests and full reporting of outcomes
☒	☐	Estimates of effect sizes (e.g. Cohen's *d*, Pearson's *r*), indicating how they were calculated

Our web collection on statistics for biologists contains articles on many of the points above.

Software and code

Policy information about availability of computer code

Data collection	Volocity 6.5, and Imaris v8 for image analysis
Data analysis	Data analysis was performed using Graphpad Prism 8.

For manuscripts utilizing custom algorithms or software that are central to the research but not yet described in published literature, software must be made available to editors/reviewers. We strongly encourage code deposition in a community repository (e.g. GitHub). See the Nature Research guidelines for submitting code & software for further information.

Data

Policy information about availability of data

All manuscripts must include a data availability statement. This statement should provide the following information, where applicable:

- Accession codes, unique identifiers, or web links for publicly available datasets
- A list of figures that have associated raw data
- A description of any restrictions on data availability

Figures 1-5, S1-3, S5 have associated raw data. Raw data are provided.

Field-specific reporting

Please select the one below that is the best fit for your research. If you are not sure, read the appropriate sections before making your selection.

☒ Life sciences ☐ Behavioural & social sciences ☐ Ecological, evolutionary & environmental sciences

For a reference copy of the document with all sections, see nature.com/documents/nr-reporting-summary-flat.pdf

Life sciences study design

All studies must disclose on these points even when the disclosure is negative.

Sample size	No statistical method was used to predetermine sample size. In each experiment, at least n=3 mice were used and at least 35 clones were analysed, in lines with the standard of the field for quantitative lineage tracing experiments.
Data exclusions	No data were excluded.
Replication	All results were replicated. Experiments were performed on at least n=3 mice for each experiment.
Randomization	The experiments were not randomized, as all animals were phenotypically wild-type, and therefore there were no covariates to control.
Blinding	The investigators were not blinded to allocation during experiments and outcome assessment. Mice used in experiments were phenotypically wild-type and the physiological process of pancreas development was studied. The quantifications were performed without a pre-formed hypothesis.

Reporting for specific materials, systems and methods

We require information from authors about some types of materials, experimental systems and methods used in many studies. Here, indicate whether each material, system or method listed is relevant to your study. If you are not sure if a list item applies to your research, read the appropriate section before selecting a response.

Materials & experimental systems

n/a	Involved in the study	
☐	☒	Antibodies
☒	☐	Eukaryotic cell lines
☒	☐	Palaeontology
☐	☒	Animals and other organisms
☒	☐	Human research participants
☒	☐	Clinical data

Methods

n/a	Involved in the study	
☒	☐	ChIP-seq
☒	☐	Flow cytometry
☒	☐	MRI-based neuroimaging

Antibodies

Antibodies used

1. Chromogranin A from Abcam, ab15160, lot no GR3229573-3
2. Dolichos biflorus agglutinin (DBA), biotinylated from Vectorlabs, B-1035, lot no ZA0417
3. Insulin from Abcam, ab7842, lot no GR3205695-1
4. Glucagon Abcam, ab10988, lot no GR3212475-1
5. Cleaved-Caspase 3 from Cell Signalling, 9664S, lot no 22
6. AF647-Streptavidin from Thermo Fisher Scientific, S21374, lot no 1812657
7. BV510-Streptavidin from Biolegend (405233), lot no B281569

Validation

1. Sznurkowska et al. 2018 Dev.Cell "Defining Lineage Potential and Fate Behavior of Precursors during Pancreas Development."; Tomic et al .2018 "Phospho-regulation of ATOH1 Is Required for Plasticity of Secretory Progenitors and Tissue Regeneration." Cell Stem Cell; antibody search website: https://www.citeab.com/antibodies/723164-ab15160-anti-chromogranin-a-antibody, Abcam website https://www.abcam.com/chromogranin-a-antibody-ab15160.html: mouse sample reactivity, application tested for ICC/IF
2. Sznurkowska et al. 2018 Dev. Cell; , Kobayashi et al 2002, "Lectin as a marker for staining and purification of embryonic pancreatic epithelium", Reichert et al, 2013, "Isolation, culture and genetic manipulation of mouse pancreatic ductal cells.".
3. Azzarelli et al. 2017 Dev. Cell "Multi-site Neurogenin3 Phosphorylation Controls Pancreatic Endocrine Differentiation."; antibody search website: https://www.citeab.com/antibodies/2274084-ab7842-anti-insulin-antibody?des=82B12C3D4761ADC2; Abcam website https://www.abcam.com/insulin-antibody-ab7842.html: mouse sample reactivity, application tested for ICC/IF
4. Azzarelli et al. 2017 Dev. Cell "Multi-site Neurogenin3 Phosphorylation Controls Pancreatic Endocrine Differentiation."; antibody search website: https://www.citeab.com/antibodies/733352-ab10988-anti-glucagon-antibody-k79bb10?des=E7C8EBB921FFFD05; Abcam website https://www.abcam.com/glucagon-antibody-k79bb10-ab10988.html: mouse sample reactivity, application tested for ICC/IF
5. Nava et al. 2020 Cell "Heterochromatin-Driven Nuclear Softening Protects the Genome against Mechanical Stress-Induced Damage"; Volta et al. 2019 Nat. Comms, "Glucose homeostasis is regulated by pancreatic β-cell cilia via endosomal EphA-processing"
6. Sznurkowska et al. 2018 Dev. Cell
7. Ho et al. 2019 Ho et al "Remodeling of Bone Marrow Hematopoietic Stem Cell Niches Promotes Myeloid Cell Expansion during Premature or Physiological Aging"; Adachi et al. 2019 Nat. Comms "Exposure of an occluded hemagglutinin epitope drives selection of a class of cross-protective influenza antibodies"

Animals and other organisms

Policy information about studies involving animals; ARRIVE guidelines recommended for reporting animal research

Laboratory animals	Mus musculus, males and females, The pups were entered into experiment at E9.5, E12.5, E14.5, E15.5, E18.5 and collected at / E18.5, P14 and P28 mice, C57BL/6 strain
Wild animals	No wild animals have been used.
Field-collected samples	No field collected samples have been used.
Ethics oversight	This research has been regulated under the Animals (Scientific Procedures) Act 1986 Amendment Regulations 2012 following ethical review by the University of Cambridge Animal Welfare and Ethical Review Body (AWERB) for Rosa26-CreERT2 lineage tracing; and the approval of the Institutional Animal Care and Use Committee of National Institutes of Natural Sciences, Japan, for Ngn3-CreERT2 lineage tracing.

Note that full information on the approval of the study protocol must also be provided in the manuscript.
